# An in vivo screening platform identifies senolytic compounds that target *p16^INK4a+^* fibroblasts in lung fibrosis

**DOI:** 10.1172/JCI173371

**Published:** 2024-03-07

**Authors:** Jin Young Lee, Nabora S. Reyes, Supriya Ravishankar, Minqi Zhou, Maria Krasilnikov, Christian Ringler, Grace Pohan, Chris Wilson, Kenny Kean-Hooi Ang, Paul J. Wolters, Tatsuya Tsukui, Dean Sheppard, Michelle R. Arkin, Tien Peng

**Affiliations:** 1 Department of Medicine, Division of Pulmonary, Critical Care, Allergy, and Sleep,; 2 Small Molecule Discovery Center, and; 3 Bakar Aging Research Institute, UCSF, San Francisco, California, USA.

**Keywords:** Aging, Pulmonology, Cellular senescence, Drug screens, Fibrosis

## Abstract

The appearance of senescent cells in age-related diseases has spurred the search for compounds that can target senescent cells in tissues, termed senolytics. However, a major caveat with current senolytic screens is the use of cell lines as targets where senescence is induced in vitro, which does not necessarily reflect the identity and function of pathogenic senescent cells in vivo. Here, we developed a new pipeline leveraging a fluorescent murine reporter that allows for isolation and quantification of *p16^Ink4a+^* cells in diseased tissues. By high-throughput screening in vitro, precision-cut lung slice (PCLS) screening ex vivo, and phenotypic screening in vivo, we identified a HSP90 inhibitor, XL888, as a potent senolytic in tissue fibrosis. XL888 treatment eliminated pathogenic *p16^Ink4a+^* fibroblasts in a murine model of lung fibrosis and reduced fibrotic burden. Finally, XL888 preferentially targeted *p16^INK4a-hi^* human lung fibroblasts isolated from patients with idiopathic pulmonary fibrosis (IPF), and reduced *p16^INK4a+^* fibroblasts from IPF PCLS ex vivo. This study provides proof of concept for a platform where *p16^INK4a+^* cells are directly isolated from diseased tissues to identify compounds with in vivo and ex vivo efficacy in mice and humans, respectively, and provides a senolytic screening platform for other age-related diseases.

## Abstract 2

A senolytic screening platform provides scalability and validation by leveraging the ability to isolate and track senescent cells in diseased tissues.

## Graphical abstract

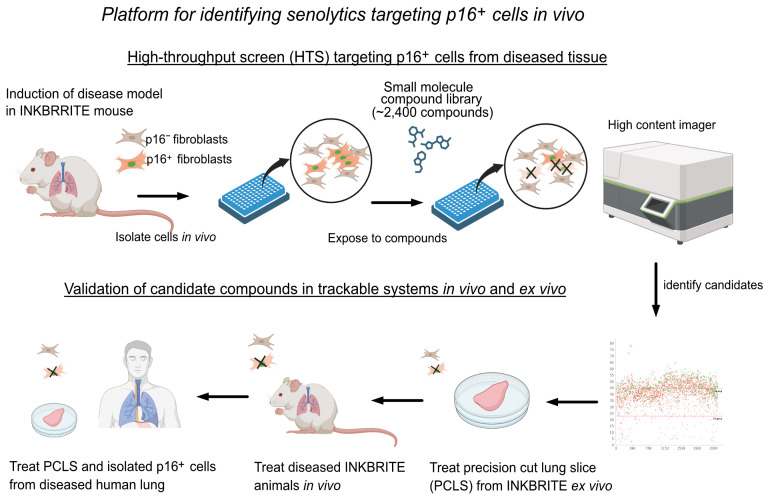

## Introduction

Hayflick’s original description of a stereotyped proliferative arrest for primary cells in culture as “senescence at the cellular level” (1) gave way to geroscience that seeks to unravel the relationship between cellular aging and age-related diseases (2). While initial studies of senescence mostly occurred in cell culture, the identification of biomarkers in vitro was extrapolated to study in vivo phenomena in aging tissues, exemplified by the use of *p16^INK4a^* expression as a biomarker of senescent cells in tissues (3). The construction of mouse models where *p16^Ink4a+^* cells can be genetically depleted to ameliorate a host of age-related phenotypes validated this approach (4–9) and identified senescent cells in tissues as potential targets for pharmacotherapy (10). The pipeline to identify compounds targeting senescent cells, termed senolytics, involves screening cytotoxicity in cell lines where senescence is induced in vitro, followed by validation in preclinical animal models where senescent cells are thought to be present (11). However, this approach assumes that senescent cells derived from cell lines in culture are identical to senescent cells in vivo that reside in far more diverse cellular environments.

Single cell transcriptome analyses have demonstrated that senescent phenotypes in vitro are heterogeneous and dependent on senescence inducers and cell types (12, 13). Based on these studies, senescent cells in vivo would be expected to display even more functional heterogeneity given the diverse tissue microenvironment they would encounter. Leveraging an ultrasensitive GFP reporter of *p16^Ink4a^* (Ink4a-H2B-GFP reporter-in-tandem, or INKBRITE), we recently demonstrated that *p16^Ink4a+^* fibroblasts in vivo exhibit a spectrum of senescent phenotypes that directly correlated with the range of *p16^Ink4a^* transcript levels (14). The recognition that senescent cells are functionally heterogeneous has profound implications for senolytic screening pipelines, as target cell type selection could significantly influence the candidates identified. This has already become apparent as different senolytic screens have identified compounds that have nonoverlapping efficacy against different cell types and preclinical models (15). To improve the ability to identify senolytics with in vivo efficacy against the disease of interest, we set out to develop a screening platform that both utilizes senescent cells directly isolated from diseased tissues as screening targets and provides an in vivo platform for direct validation of senolytic activity in the tissue of origin.

Idiopathic pulmonary fibrosis (IPF) is an age-related lung disease where telomere shortening and senescence have been implicated in the pathogenesis (16, 17). Histologic examination of IPF lungs has demonstrated the presence of biomarkers associated with senescence in alveolar type 2 (AT2) cells and lung fibroblasts (18), which was confirmed in single cell atlases of IPF lungs showing enrichment of *CDKN2A* (encoding *p16^INK4a^*) expression in epithelial and fibroblast subsets (19, 20). Despite histologic evidence of p16^INK4a+^ cells in IPF and animal models of lung fibrosis, their pathogenic role is less clear. Genetic ablation of *p16^Ink4a+^* cells in an animal model of lung fibrosis improved lung function, but there was no evidence that this animal model improved standard fibrotic endpoints such as hydroxyproline content (21). Furthermore, genetic depletion studies preclude the ability to identify *p16^Ink4a+^* cells in the fibrotic tissue and study their behavior in a prospective manner. To determine whether *p16*^Ink4a+^ cells are a viable target for therapeutic intervention in lung fibrosis, we need to isolate and characterize these cells in vivo. In this study, we characterized the identity of fibrotic *p16^Ink4a+^* fibroblasts in vivo and outlined a senolytic screening platform that provides scalability and validation by leveraging the ability to isolate and track *p16^Ink4a+^* cells from diseased tissues. Utilizing our new platform, we identified a novel senolytic compound that deleted *p16^Ink4a+^* cells, reduced fibrotic burden in a murine model of lung fibrosis, and preferentially targeted *p16^Ink4a+^* fibroblasts from human IPF lung samples.

## Results

### Murine p16^Ink4a+^ lung fibroblasts display profibrotic identities and phenotypes in vivo.

**Figure 1:**
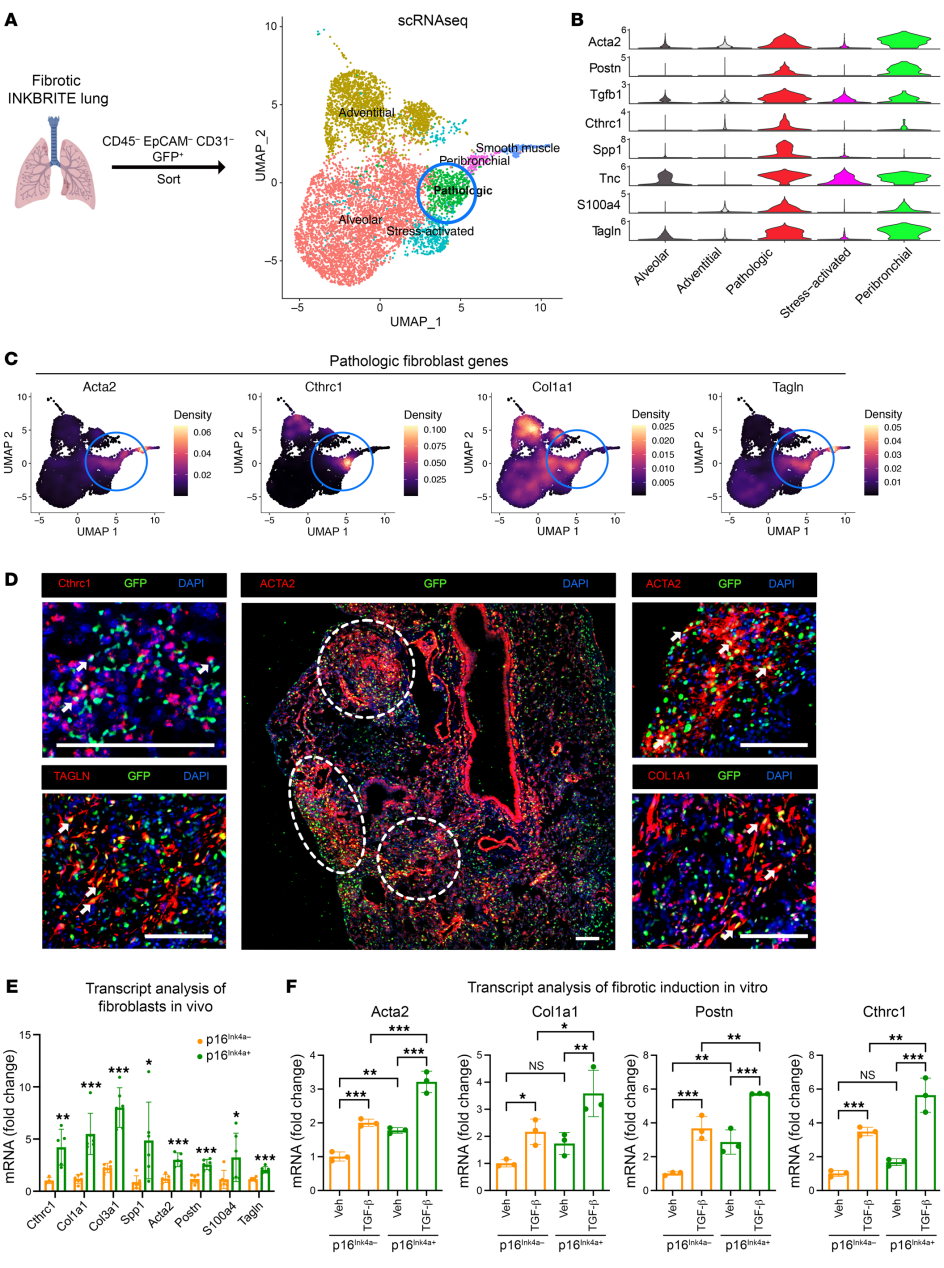
*p16^Ink4a+^* fibroblasts contribute to pathologic fibroblasts in mouse model of lung fibrosis. (**A**) Experimental scheme for scRNA-Seq of *p16^Ink4a+^* (GFP^+^) fibroblasts from the INKBRITE lung after bleomycin-induced fibrosis (14 dpi). (**B**) Violin plot showing profibrotic gene expressions in the different *p16^Ink4a+^* fibroblast subsets in vivo. (**C**) Visualization of *Acta2*, *Cthrc1*, *Col1a1*, and *Tagln* expression pattern within the fibroblast subsets. (**D**) Representative images showing *Cthrc1* (RNAscope in situ), ACTA2, TAGLN, and COL1A1 (immunostaining) in lung sections of bleomycin-injured INKBRITE mice (14 dpi) colocalized with GFP (arrows, *p16^Ink4a+^* fibroblasts). Scale bars: 100 μm. (**E**) qPCR analysis of purified GFP^+^ and GFP^–^ fibroblasts from bleomycin-treated INKBRITE lungs (*n* = 5–6 biological replicates, experiment repeated twice). (**F**) qPCR analysis of cultured GFP^+^ and GFP^–^ fibroblasts isolated from fibrotic INKBRITE lungs after treatment of recombinant TGF-β1 or vehicle (*n* = 3 technical replicates, experiment repeated twice). Data are represented as mean ± SD. *P < 0.05; **P < 0.01; ***P < 0.001; 2-tailed Student’s *t* test (**E**) or 1-way ANOVA (**F**).

Our previous study showed that *p16^Ink4a+^* fibroblasts dynamically respond to tissue injury in the lung (14). To define the potential contribution of *p16^Ink4a+^* fibroblasts to fibrogenesis, we performed single cell RNA-Seq (scRNA-Seq) on fibrotic INKBRITE lungs. Leveraging our capacity to isolate *p16^Ink4a+^* cells by fluorescent sorting, we isolated GFP^+^ fibroblasts (CD45^–^ EpCAM^–^ CD31^–^) from lungs of INKBRITE mice treated with bleomycin (14 days postinjury, or dpi). We profiled 7,846 cells and identified 6 clusters with distinct marker gene expressions that corresponded with previously annotated fibroblast subsets identified in fibrotic murine lungs (22, 23) (Figure 1A and Supplemental Figure 1, A and B; supplemental material available online with this article; https://doi.org/10.1172/JCI173371DS1). In addition to the adventitial, alveolar, and peribronchial fibroblast subsets that were previously identified in the uninjured lungs, we observed significant fractions of pathologic (expressing numerous profibrotic genes) and stress-activated fibroblasts that were found to arise de novo in the fibrotic lung (Figure 1, A–C) (22, 23). These pathologic fibroblasts were mostly absent from our prior single cell data set of *p16^Ink4a+^* fibroblasts isolated from naphthalene-injured INKBRITE lungs (14) (Supplemental Figure 1, A–E), showing that the lineage fate of *p16^Ink4a+^* fibroblasts changes with the injury context. IHC of injured INKBRITE lungs demonstrated infiltration of GFP^+^ fibroblasts within dense, fibrotic regions (Figure 1D, dashed circles). IHC analysis validated the scRNA-Seq results, demonstrating the presence of *p16^Ink4a+^*/*Cthrc1*^+^ fibroblasts within the fibrotic regions that also contain other fibrotic markers such as ACTA2, COL1A1, and TAGLN (Figure 1D).

To determine phenotypic differences between *p16^Ink4a–^* and *p16^Ink4a+^* fibroblasts in fibrotic tissue, we FACSorted GFP^+^ and GFP^–^ fibroblasts from bleomycin-injured INKBRITE lungs for downstream analyses. Quantitative PCR (pPCR) confirmed the upregulation of *p16^Ink4a^* as well as p21 in GFP^+^ fibroblasts, along with phenotypic markers associated with senescence such as cell size, DNA damage, and proliferative arrest (Supplemental Figure 2, A–E). Further, *p16^Ink4a+^* fibroblasts demonstrated significant upregulation for genes enriched in pathologic fibroblasts compared with *p16^Ink4a–^* fibroblasts (Figure 1E), which was confirmed on immunocytochemistry of sorted GFP^+^ and GFP^–^ fibroblasts from fibrotic INKBRITE lungs (Supplemental Figure 3, A–C). To investigate potential differences in response to fibrotic stimuli between *p16^Ink4a+^* and *p16^Ink4a–^* cells, we isolated GFP^+^ and GFP^–^ lung fibroblasts from uninjured INKBRITE lungs and stimulated them with recombinant TGF-β1. *p16^Ink4a+^* fibroblasts exhibited elevated fibrotic gene expression both before and after TGF-β1 stimulation compared with *p16^Ink4a–^* fibroblasts (Figure 1F). These data indicate that *p16^Ink4a+^* lung fibroblasts preferentially give rise to pathologic fibroblasts in lung fibrosis. More importantly, our data suggest that different types of *p16^Ink4a+^* fibroblasts arise in different pathologic contexts, which could also dictate divergent susceptibility of *p16^Ink4a+^* cells to senolytics under different tissue conditions.

### p16^Ink4a^ expression in lung fibroblasts augments the fibrotic response.

**Figure 2:**
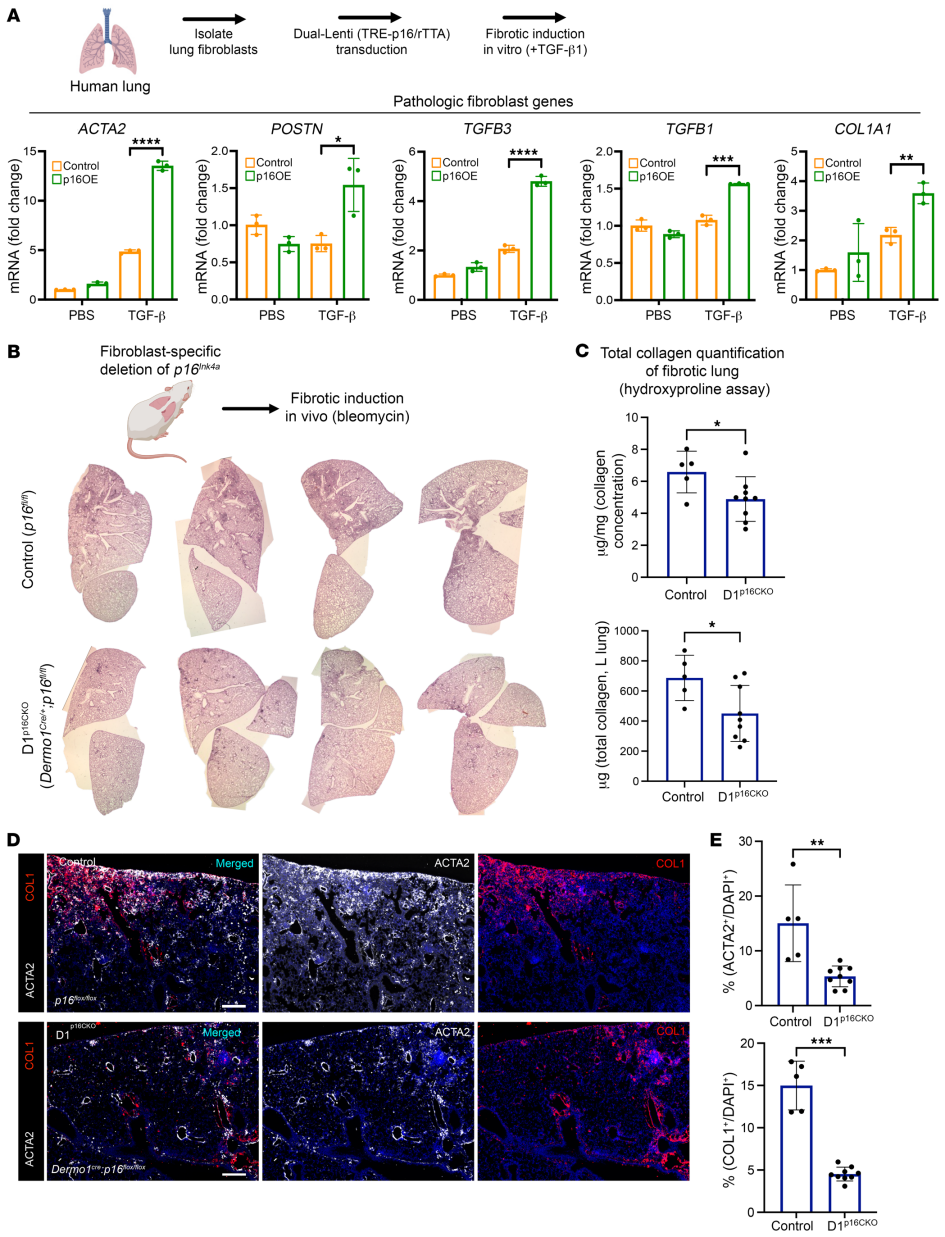
*p16^INK4a^* expression primes lung fibroblasts to augment the fibrotic response. (**A**) Transcript analysis of cultured primary human lung fibroblasts isolated from control cadaveric donors transduced with 2 lentiviral vectors to overexpress (OE) human *p16^INK4a^* with doxycycline induction followed by addition of TGF-β1. (*n* = 3 technical replicates, experiment repeated twice). (**B**) Representative H&E sections of *Dermo1^Cre/+^;p16^fl/fl^* and control (*p16^fl/fl^*) animals injured with bleomycin to induce lung fibrosis. (**C**) Hydroxyproline assay to quantify collagen in the left lung of *Dermo1^Cre/+^;p16^fl/fl^* and control animals 14 days following bleomycin injury (*n* = 5 control, 9 mutant biological replicates). (**D**) Representative IHC showing ACTA2 and COL1 immunostaining in lung sections of bleomycin-injured *Dermo1^Cre/+^;p16^fl/fl^* and control (*p16^fl/fl^*) animals (14 dpi). (**E**) IHC quantification of ACTA2^+^ and COL1^+^ fibroblasts from **D** (*n* = 5 control, 9 mutant biological replicates. Scale bars: 200 μm. Data are represented as mean ± SD. *P < 0.05; **P < 0.01; ***P < 0.001; ****P < 0.0001; 2-tailed Student’s *t* test (**A**, **C**, and **E**).

To determine the role of *p16^INK4a^* expression in the fibrotic response, we first generated a dual lentiviral system (Lenti-tTS/rtTA + Lenti-TRE-p16-T2A-dTomato) to overexpress *p16^INK4a^* in a doxycycline-dependent manner (14). Primary human lung fibroblasts isolated from control cadaveric donors were transduced with our *p16^INK4a^* overexpression (OE) vectors, followed by doxycyline and fibrotic induction in vitro (addition of TGF-β1). *p16^INK4a^* OE significantly augmented the expression of pathologic fibroblast genes in response to TGF-β1 compared with control fibroblasts (Figure 2A). Interestingly, *p16^INK4a^* OE in the absence of TGF-β1 did not induce profibrotic gene expression (Figure 2A), suggesting that *p16^INK4a^* alone does not independently drive the fibrotic response, but rather primes the fibroblast response to a fibrotic stimulus.

To determine the necessity of *p16^Ink4a^* expression in the fibroblast response to fibrotic stimuli in vivo, we deleted *p16^Ink4a^* in fibroblasts with a mesenchymal-specific Cre-driver (*Dermo1^Cre/+^*). As we had previously reported, fibroblast-specific deletion of *p16^Ink4a^* (*Dermo1^Cre/+^:p16^fl/fl^*, referred to as *D1^p16CKO^*) did not alter the gross morphology in the uninjured lung (14). Induction of fibrotic injury with bleomycin demonstrated that *D1^p16CKO^* animals exhibited attenuated fibrotic response on histology and collagen deposition (as measured by hydroxyproline) compared with controls (Figure 2, B and C). IHC analysis demonstrated that fibroblast-specific deletion of *p16^Ink4a^* reduced the number of fibroblasts expressing profibrotic markers in the bleomycin-injured lungs (Figure 2, D and E). These experiments demonstrated that *p16^Ink4a^* expression primes the fibroblasts to augment the fibrotic response but is not sufficient to drive fibrosis in the absence of a fibrotic stimulus.

### High throughput screen identified compounds targeting p16^Ink4a+^ lung fibroblasts isolated from fibrotic tissue.

**Figure 3:**
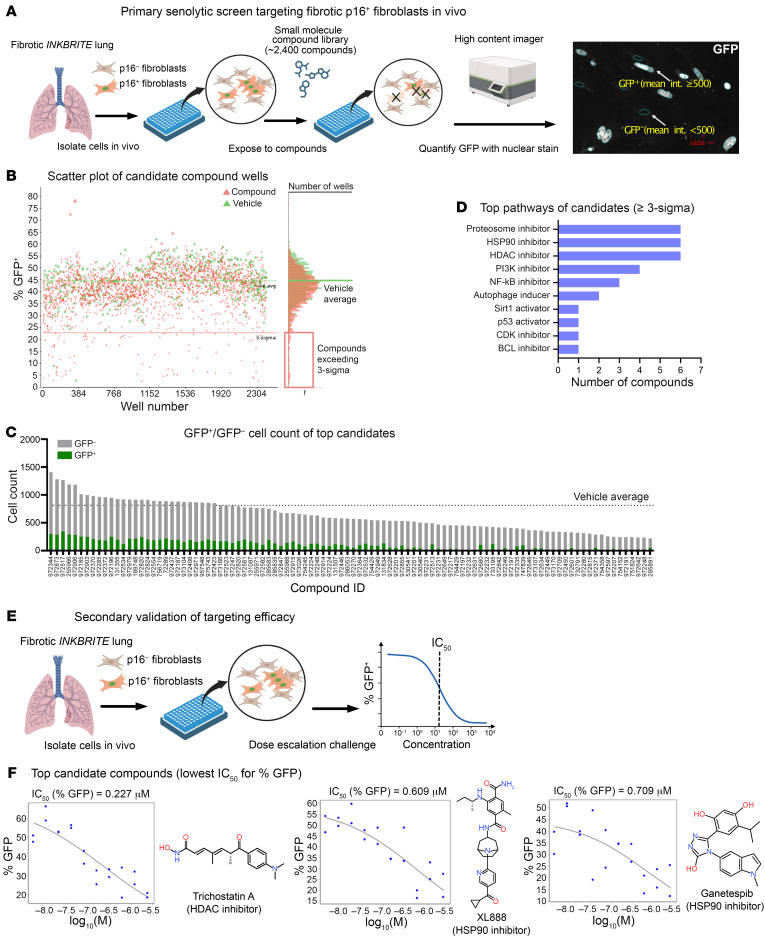
HTS targeting *p16^Ink4a+^* fibroblasts isolated from fibrotic INKBRITE lungs. (**A**) Schematic outline of the HTS to identify compounds targeting *p16^Ink4a+^* (GFP^+^) fibroblasts from the fibrotic INKBRITE lungs. (**B**) Scatter plot showing hit results from each well containing compound (pink) or vehicle (green). Y-axis indicates %GFP^+^ cells in each well after compound exposure. Compounds exceeding 3 σ for lowest %GFP were selected for validation. (**C**) Cell count GFP^+^ and GFP^–^ fibroblasts of the top senolytic candidates. (**D**) Biologic pathways targeted by the top senolytic candidates. (**E**) Schematic outline of dose-response analysis of the top senolytic candidate from the primary screen. (**F**) Top candidates emerging from the secondary validation using dose-response with lowest IC_50_ values, including trichostatin A, XL888, and ganetespib.

Proper target selection is one of the most important factors for successful compound screens. To identify compounds that will specifically target *p16^Ink4a+^* fibroblasts from fibrotic lungs, we leveraged the ability to isolate *p16^Ink4a+^* fibroblasts in vivo from diseased tissues utilizing the INKBRITE reporter. We purified *p16^Ink4a+^* (GFP^+^) and *p16^Ink4a–^* (GFP^–^) fibroblasts in vivo directly from the fibrotic lungs of INKBRITE animals injured with bleomycin (14 dpi). The fluorescent tag allowed us to mix GFP^+^ and GFP^–^ fibroblasts at a 1:1 ratio into 384-well plates, so that each well could serve as an internal control when comparing the cell viability of *p16^Ink4a+^* and *p16^Ink4a–^* fibroblasts. The ability to combine the target (*p16^Ink4a+^*) and bystander (*p16^Ink4a–^*) cells together in the same well also allowed us to scale up the screen to a chemical library of roughly 2,000 small molecules with annotated biologic activity (Figure 3A). The goal of the primary screen was to identify the most potent compounds that killed off GFP^+^ fibroblasts while sparing GFP^–^ fibroblasts as determined by the percentage of GFP^+^ fibroblasts (%GFP^+^) in each well. The fluorescent intensity of the INKBRITE reporter and nuclear localization of H2B-GFP allowed image segmentation of GFP^+^ and GFP^–^ nuclei in over 2,600 wells with high content imaging, and we identified 37 compounds that exceeded the statistical threshold of 3 σ (< 20%GFP^+^) from the mean (45% GFP^+^ in negative control/vehicle wells) (Figure 3, B and C). Analysis of the annotated biological pathways demonstrated numerous pathways previously implicated in senolysis (24) (e.g., HSP90, BCL-2, PI3K inhibitors) as well as potentially novel ones (e.g., HDAC, proteasome inhibitor) (Figure 3D). Of note, previously identified senolytics such as dasatinib, quercetin, and fisetin all reduced %GFP^+^ below the mean, but none exceeded the 3-σ threshold for secondary validation (Supplemental Table 1).

To define the potency of these compounds to delete *p16^Ink4a+^* cells, we selected 32 compounds with the lowest %GFP^+^ for secondary validation with dose-response curves to determine the half-maximal inhibitory concentration (IC_50_) for reduction of %GFP^+^ fibroblasts (Figure 3E). Each compound was tested over 10 concentrations to a maximum of 20 mM. The validation screen yielded 8 compounds that had an IC_50_ (%GFP) below 2 μM, which was composed mostly of HSP90 and HDAC inhibitors (Supplemental Table 2). The top candidates typically had a maximal effect (E_max_) of reducing GFP^+^ fibroblast viability < 10% (Supplemental Figure 4). The top 3 candidates had IC_50_ (reduction of %GFP) below 1 μM, including Trichostatin A (TSA, HDAC inhibitor), XL888 (heat shock protein 90, or HSP90 inhibitor), and Ganetesipib (HSP90 inhibitor) (Figure 3F).

### An ex vivo model using PCLS to validate senolytic candidates.

**Figure 4:**
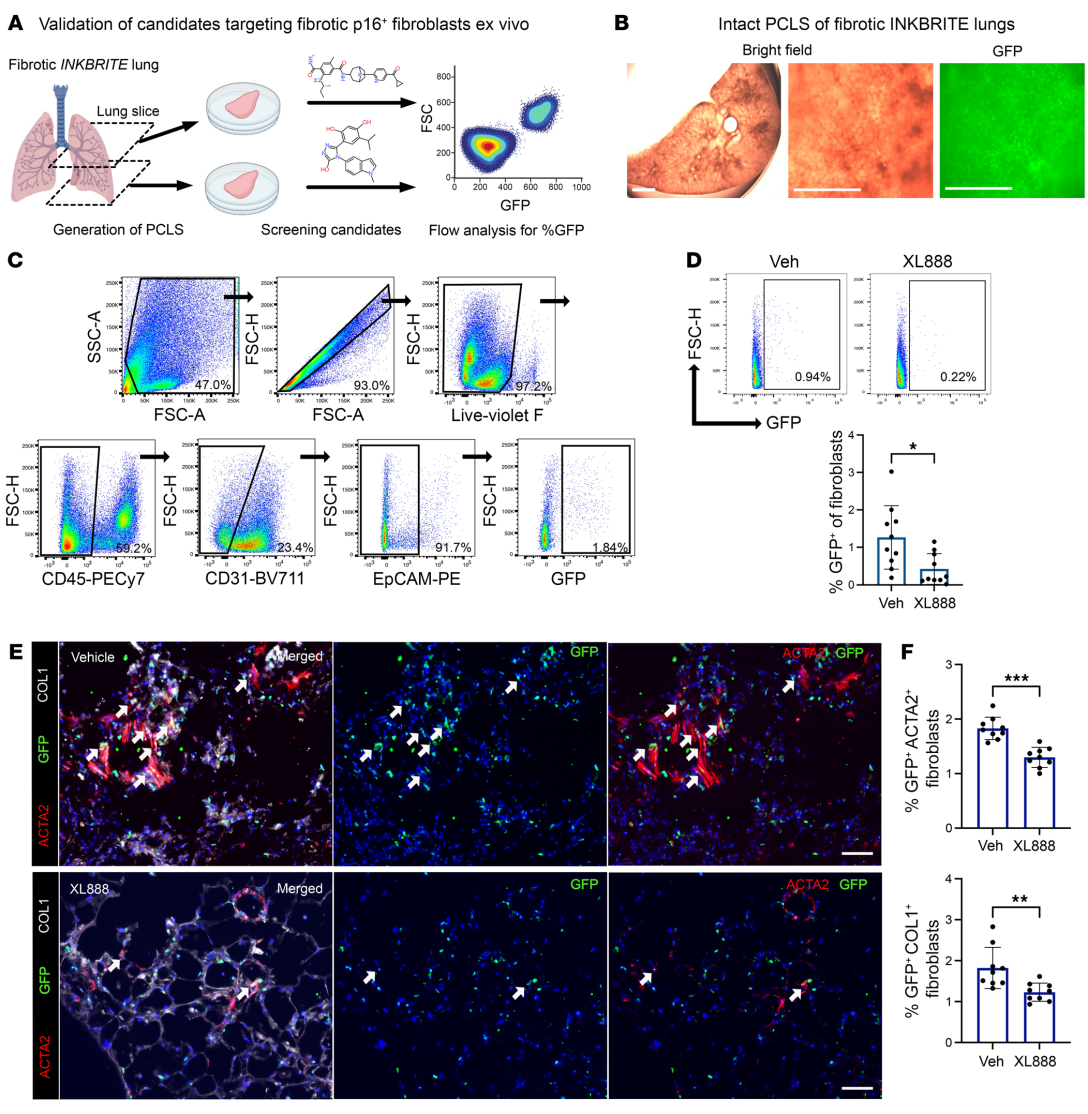
Validation of candidate senolytic compounds using mouse PCLS derived from fibrotic INKBRITE lungs. (**A**) Experimental scheme for ex vivo culture of mouse PCLS derived from fibrotic INKBRITE mouse to test senolytic candidates. (**B**) Bright field and GFP images of cultured PCLS. Scale bars: 2,000 μm. (**C**) Gating strategy to analyze GFP^+^ fibroblasts from mouse PCLS by flow cytometry. (**D**) Quantification of GFP^+^ fibroblasts in the PCLS cultured with vehicle or XL888 (1 μM) for 5 days (*n* = 10 technical replicates, experiment repeated twice). (**E** and **F**) Immunofluorescence analysis (**E**) and quantification (**F**) of ACTA2, COL1A1, and GFP in mouse PCLS treated with vehicle or XL888 (1 μM). (*n* = 9 technical replicates, experiment repeated twice). Scale bars: 50 μm. Data are represented as mean ± SD. *P < 0.05; **P < 0.01; ***P < 0.001; 2-tailed Student’s *t* test (**D**, **F**).

A bottleneck to the compound screening pipeline is narrowing the top hits that are most likely to be efficacious in animal disease models, which can be expensive and time consuming to perform. To predict in vivo efficacy to streamline our screen, we incorporated an ex vivo platform to test the top hits in our screen in a more physiologic setting that is easily scalable. Ex vivo culture of precision-cut lung slices (PCLSs) has been utilized to model lung diseases such as IPF as well as drug testing for lung fibrosis (25). We first generated PCLS cultures from bleomycin-injured INKBRITE animals (Figure 4, A and B). Flow cytometry analysis demonstrated high viability of the cells in the PCLS at the end of the 5-day culture period (Supplemental Figure 5A). We tested the top candidates identified in the secondary validation in the fibrotic INKBRITE PCLS ex vivo using the same standard drug concentration as the primary screen. Again, we wanted to examine the effect of the compound on *p16^Ink4a+^* fibroblasts relative to *p16^Ink4a–^* fibroblasts, so we examined the %GFP in the fibroblast population (CD45^–^/EpCAM^–^/CD31^–^; Figure 4C) on flow analysis of the treated PCLS. Flow analysis demonstrated that both HSP90 inhibitors (XL888 and ganetespib) reduced the %GFP^+^ fibroblasts in the INKBRITE PCLS, while TSA had no effect (Figure 4D and Supplemental Figure 5B). Histology of INKBRITE PCLS demonstrated preserved architecture and continued presence of ACTA2^+^/*p16^Ink4a+^* and COL1^+^/*p16^Ink4a+^* fibroblasts in the fibrotic regions of the lung (Figure 4E). IHC analysis of XL888-treated PCLS demonstrated a reduction of GFP^+^ACTA2^+^ and GFP^+^COL1^+^ fibroblasts (Figure 4, E and F). We also screened 2 other HDAC inhibitors with low IC_50_ in the dose-response validation in vitro (fimepinostat and dacinostat), but neither compound reduced %GFP^+^ fibroblasts ex vivo in our PCLS assay (Supplemental Figure 5C). Finally, we tested other previously described senolytics in our PCLS model (dasatinib + quercetin or D&Q, fisetin, ABT-263 and ABT-737) in comparison with XL888, and only XL888 reduced %GFP^+^ fibroblasts compared with vehicles (Supplemental Figure 5D). GFP over-expression in normal lung fibroblasts did not enhance susceptibility to XL888-mediated killing (Supplemental Figure 5E). Finally, we tested XL888 on PCLS isolated from INKBRITE animals induced with a nonfibrotic injury using naphthalene (airway injury followed by full repair). In contrast to fibrotic injured PCLS, XL888 did not significantly reduce the % of GFP^+^ fibroblasts from naphthalene-injured PCLS (Supplemental Figure 5F). These experiments suggest that HSP90 inhibitors are the most promising candidates to target *p16^Ink4a+^* fibroblasts in vivo in preclinical animal models of lung fibrosis.

### XL888 deletes p16^INK4a+^ lung fibroblasts in vivo and attenuates fibrotic remodeling in mice.

**Figure 5:**
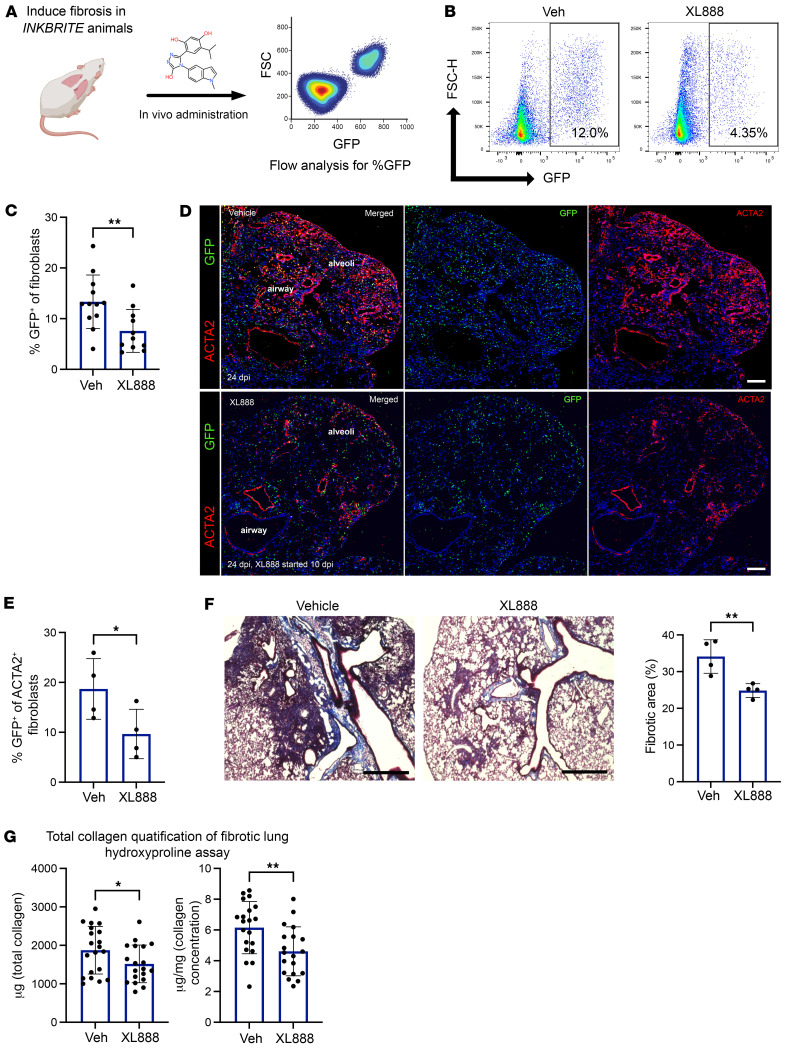
XL888 deletes *p16^Ink4a+^* fibroblasts and attenuates fibrotic remodeling in vivo. (**A**) Schematic outline of animal experiments to validate in vivo efficacy of candidate senolytics. (**B** and **C**) Flow cytometry analysis of GFP^+^ fibroblasts (% of fibroblasts that are GFP^+^) in bleomycin-injured lungs of vehicle or XL888 delivered INKBRITE animals (*n* = 11–12 biological replicates, experiment repeated twice). (**D** and **E**) Immunofluorescence analysis (**D**) and quantification (**E**) of GFP^+^ cells among ACTA2^+^ fibroblasts in the lungs of vehicle or XL888-treated INKBRITE mice (*n* = 4 biological replicates, experiment repeated twice). Scale bars: 100 μm. (**F**) Representative images (left) and quantification of Masson’s trichrome staining of lung sections from indicated group of mice after bleomycin injury (*n* = 4 biological replicates). Scale bars: 1,000 μm. (**G**) Quantitative analysis of collagen in lung homogenates from vehicle or XL888 treated animals injured with bleomycin (*n* = 19–20 biological replicates, experiment repeated twice). Data are represented as mean ± SD. *P < 0.05; **P < 0.01; 2-tailed Student’s *t* test (**C**); or 1-tailed Student’s *t* test (**E**–**G**).

To determine whether the PCLS screen correlated with in vivo activity against *p16^Ink4a+^* fibroblasts, we set up an in vivo validation step where each of the candidate compounds tested in PCLS (XL888, ganetespib, TSA, fimepinostat, and dacinostat) was administered in our preclinical model of lung fibrosis and compared against vehicle-treated control animals (each compound required a different vehicle cohort because each is formulated differently with different routes of administration). INKBRITE animals were first injured with bleomycin to establish fibrosis, followed by administration of the candidate senolytic daily for 2 weeks (Figure 5A). For each compound, we determined the maximum effective dose based on literature search of previous use in other preclinical models. At the end of the treatment period, we determined the selective impact of the compound on *p16^Ink4a+^* fibroblasts using an identical flow cytometry strategy as described for PCLS (Figure 4C). Flow analysis of single cell lung suspension at the end of the study period demonstrated that only XL888 treatment resulted in a significant reduction in the %GFP^+^ fibroblasts in vivo compared with vehicle controls (Figure 5, B and C and Supplemental Figure 6, A–D).

To determine whether XL888 targeted pathologic *p16^Ink4a+^* subsets described in our scRNA-Seq analysis, we repeated the XL888 treatment in fibrotic INKBRITE animals for histologic analysis. IHC showed that treatment of XL888 reduced the GFP^+^/ACTA2^+^ fibroblasts within fibrotic regions of the lung (Figure 5, D and E). To determine whether XL888 reduced the overall fibrotic burden in the lung, we performed trichrome staining and hydroxyproline quantification in another cohort of XL888-treated animals compared with vehicles, which showed reduction in fibrotic remodeling in the lung as well as collagen content as measured by hydroxyproline (Figure 5, F and G). Finally, we administered D&Q in our preclinical model using the same dosing schedule as previously reported (21) (3 times for a 3 week treatment period) in the INKBRITE mice, and we could not observe a difference in the %GFP^+^ fibroblasts at the end of the study period, although there was a trend toward reduction in %GFP^+^ immune and endothelial cells (Supplemental Figure 6, E–G). D&Q did not change the total collagen content by hydroxyproline assay (Supplemental Figure 6H). These results showed that XL888, identified from our HTS platform, effectively eliminated *p16^Ink4a+^* fibroblasts in vivo and attenuated pulmonary fibrosis in an animal model. The results demonstrated that *p16^Ink4a+^* fibroblasts play a functional role in the development of lung fibrosis and indicate their potential as therapeutic targets for diseases involved in the accumulation of those cells.

### Human p16^INK4a+^ fibroblasts contribute to pathologic fibroblast subsets in IPF.

**Figure 6:**
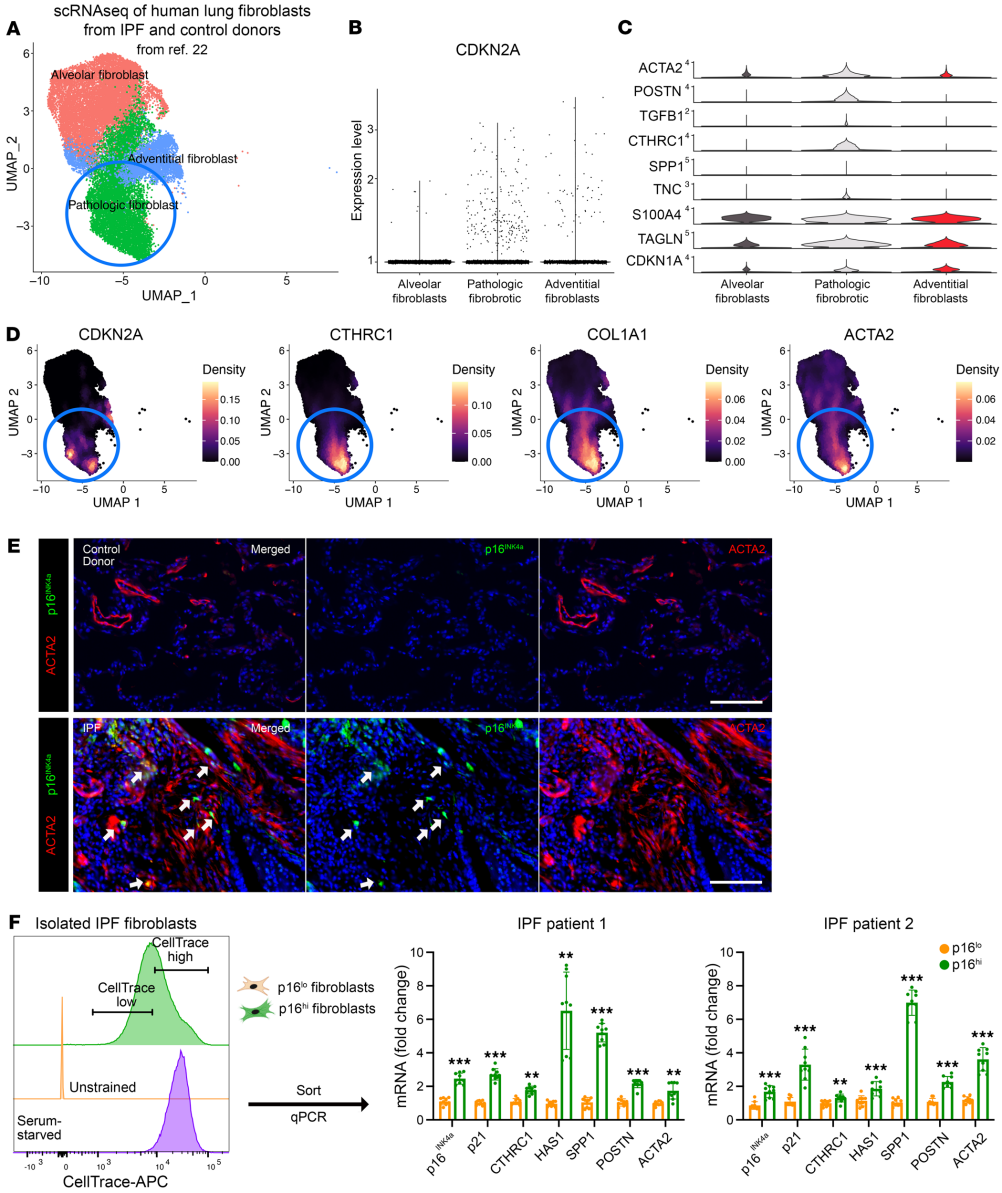
Human *p16^INK4a+^* fibroblasts contribute to pathologic fibroblasts in IPF. (**A**) UMAP plot of fibroblast subsets seen in normal human and IPF lungs. (**B**) Violin plot showing *CDKN2A* expression in the pathologic fibroblast cluster of IPF fibroblasts. (**C**) Violin plots showing the profibrotic genes in the different fibroblast subsets. (**D**) Visualization of *CDKN2A*, *CTHRC1*, *COL1A1*, and *ACTA2* expression patterns in human lung fibroblasts in IPF and control donor lungs. (**E**) Representative images showing ACTA2^+^p16^INK4a+^ pathologic fibroblasts (arrows) in lung sections of controls and individuals with IPF. Scale bars: 100 μm. (**F**) qPCR analysis of genes enriched in pathologic fibroblasts in *p16^INK4a^*^-hi^ and *p16^INK4a^*^-lo^ fibroblasts isolated from lungs of patients with IPF (*n* = 9 technical replicates, experiments repeated with separate IPF donor fibroblasts at least 3 times). Data are represented as mean ± SD. *P < 0.05; **P < 0.01; ***P < 0.001; 2-tailed Student’s *t* test (**F**).

While IPF fibroblasts cultured in vitro have been reported to demonstrate senescent characteristics (26), we sought to determine the expression pattern of *CDKN2A* (encoding both *p16^INK4a^* and *p19^ARF^*) in our previous scRNA-Seq of IPF lung fibroblasts in vivo (22) (Figure 6A and Supplemental Figure 7A). Our previous analysis of IPF fibroblasts demonstrated distinct clusters of alveolar and adventitial fibroblasts comparable with those defined in mice, along with the emergence of a *CTHRC1*^+^/*COL1A1*^hi^/*ACTA2*^hi^ pathologic fibroblast subset similar to the one that arose in murine lungs after bleomycin injury (22). Analysis of *CDKN2A* expression showed significant enrichment in the *CTHRC1*^+^/*COL1A1*^hi^/*ACTA2*^hi^ fibroblast subset that had been found to form fibroblastic foci in the IPF lungs (Figure 6, B–D). qPCR of IPF fibroblasts demonstrated significantly higher *p16^INK4a^* expression compared with lung fibroblasts isolated from normal controls (Supplemental Figure 7B; patient demographic data in Supplemental Table 3). IHC analysis of IPF lungs demonstrated the presence of *p16^Ink4a+^*/ACTA2^+^ and *p16^Ink4a+^*/*CTHRC1*^+^ cells within areas of dense fibrotic remodeling and the absence of these cells in the normal control lungs (Figure 6E, Supplemental Figure 7C).

We previously reported a technique to isolate senescent *p16^INK4a^*^-hi^ fibroblasts from the human lung by pulsing human lung fibroblasts with CellTrace Far Red (CT^FR^), a fluorescent dye that is diluted with cell division, and isolating CT^FR^-retaining (CT^FR-hi^) cells that developed proliferative arrest in culture (14). We applied this technique to segregate CT^FR-hi^ and CT^FR-lo^ fibroblasts from IPF lungs (from patients undergoing lung transplantation), and qPCR demonstrated significant enrichment of *p16^INK4a^* expression in the CT^FR-hi^ population (but not *p14^ARF^* encoded in the same locus) along with other pathologic fibroblast markers such as *p21*, *CTHRC1*, *HAS1*, and *PostN* (Figure 6F, Supplemental Figure 7D). These results demonstrate that profibrotic IPF lung fibroblasts are enriched for *p16^INK4a^* expression, and they can be isolated for drug testing.

### XL888 deletes human p16^INK4a+^ fibroblasts from IPF lungs.

**Figure 7:**
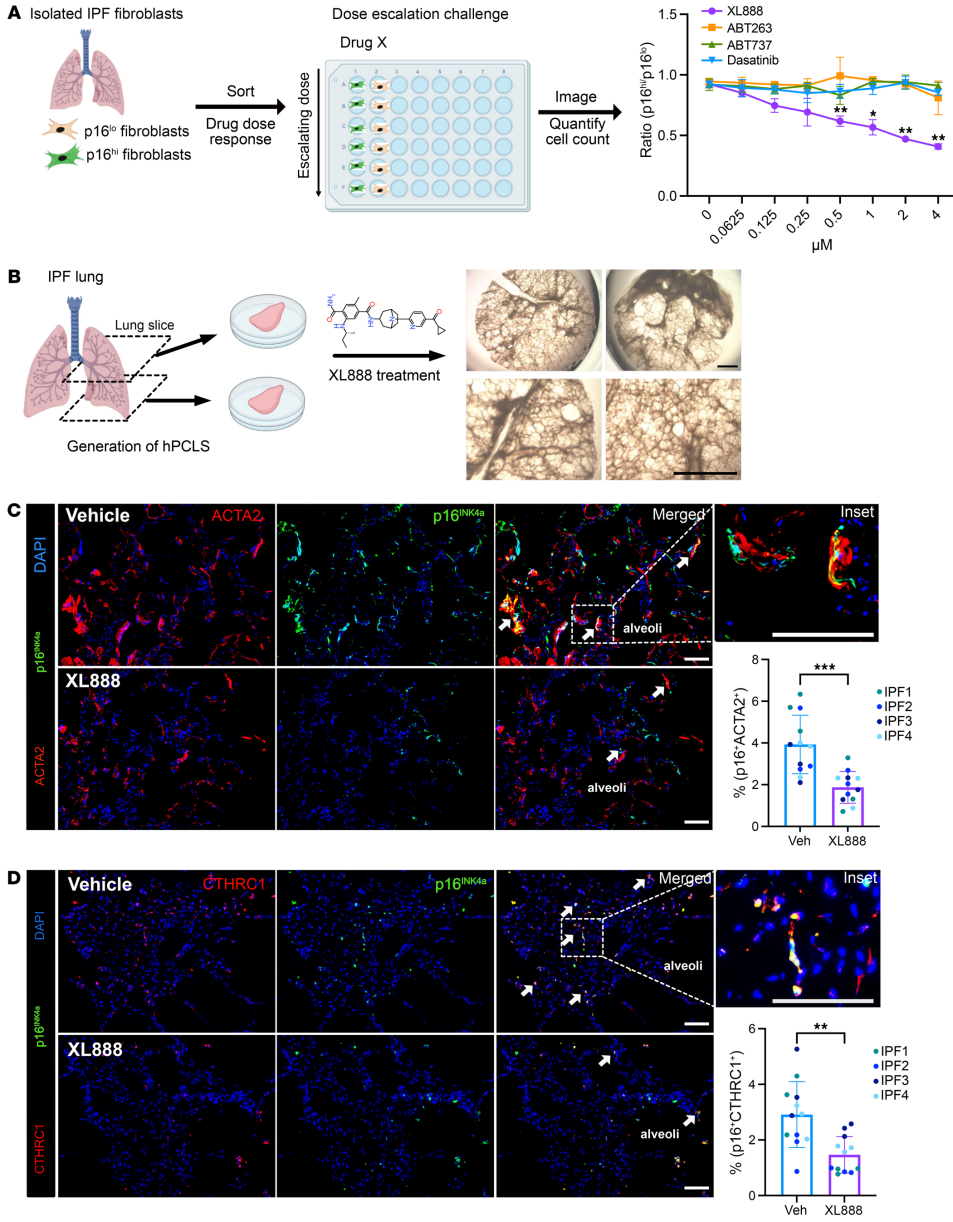
XL888 targets human *p16^INK4a+^* fibroblasts from IPF lungs in vitro and ex vivo. (**A**) (Left) Schematic outline of dose-escalation challenge of candidate senolytics on *p16^INK4a^*^-hi^ and *p16^INK4a^*^-lo^ fibroblasts isolated from IPF lungs (Right) Ratio of *p16^INK4a^*^-hi^ and *p16^INK4a^*^-lo^ fibroblast cell count after treatment of senolytics and XL888 with dose escalation (*n* = 3 technical replicates, experiments repeated with separate IPF donor fibroblasts at least 3 times). (**B**) Schematic diagram depicting ex vivo culture of IPF lung with XL888 treatment and bright field images of cultured human PCLS. Scale bars: 2,000 μm. (**C**) Immunofluorescence analysis and quantification of ACTA2^+^ p16^INK4a+^ cells in vehicle or XL888-treated hPCLS (*n* = 12 slices per condition, sampled from 4 IPF donors independently, each color represents a different donor). Scale bars: 100 μm. (**D**) Immunofluorescence analysis and quantification of CTHRC1^+^ p16^INK4a+^ cells in vehicle or XL888-treated hPCLS (*n* = 12 slices per condition, sampled from 4 IPF donors independently, each color represents a different donor). Scale bars: 100 μm. Data are represented as mean ± SD. *P < 0.05; **P < 0.01; ***P < 0.001; 2-tailed Student’s *t* test (**A**, **C**, and **D**).

To determine whether XL888 preferentially targets *p16^INK4a^*^-hi^ relative to *p16^INK4a^*^-lo^ fibroblasts from human lungs, we sorted CT^FR-hi^ and CT^FR-lo^ fibroblasts isolated from explanted IPF lungs. CT^FR-hi^ and CT^FR-lo^ fibroblasts were sorted into separate wells and dose-escalation challenge with XL888 was performed along with other previously identified senolytics (ABT263, ABT737, and dasatinib) to determine their potency in deleting *p16^INK4a^*^-hi^ versus *p16^INK4a^*^-lo^ fibroblasts. Treatment of XL888 preferentially deleted CT^FR-hi^ fibroblasts compared with CT^FR-lo^ IPF fibroblasts (Figure 7A). In contrast, previously described senolytic compounds did not exhibit selective targeting of CT^FR-hi^ fibroblasts, similar to what we found in the PCLS derived from INKBRITE murine lungs (Supplemental Figure 5D). To investigate the potential efficacy of XL888 in IPF ex vivo, we utilized PCLS generated from IPF lung tissues (Figure 7B). Flow cytometry analysis indicated high viability of the cells in the PCLS at the end of the 5-day culture period (Supplemental Figure 7E). IHC analysis of human PCLS showed that XL888 treatment decreased pathologic p16^INK4a+^ACTA2^+^ and p16^INK4a+^CTHRC1^+^ fibroblasts (Figure 7, C and D). Taken together, these results demonstrate that our senolytic HTS identified a compound that preferentially targets *p16^Ink4a+^* fibroblasts from fibrotic lungs, which was subsequently validated in vivo and ex vivo in mouse models of lung fibrosis and human IPF samples, respectively.

## Discussion

The success of high-throughput screens (HTS) of small molecules, as defined by the identification of compounds that are effective in vivo, is highly dependent on whether the screening target approximates the physiological target that will ultimately serve to validate the discovery (27). Molecular and cellular targets are selected based on their relevance to the disease state, as well as the ease with which these targets can be obtained. Over the past decade, remarkable progress has been made on the identification of senolytics, or compounds targeting cells with senescent properties that have been implicated in age-related pathologies. Hypothesis-driven candidate screens that identified senolytics such as D&Q, navitoclax, and fisetin have been shown to display in vivo efficacy in disease models (10, 28, 29); however, the cellular targets for these senolytics are not redundant. Utilizing various human cell lines where senescence is induced in vitro, these early studies showed that different senolytics have cell-type specific efficacy in vitro, which accounts for the difference in their efficacy in different mouse disease models in vivo. This suggested a heterogeneity in the mechanisms adopted by senescent cells to resist apoptosis and highlighted the need for a more rational approach where the screening target cell type directly approximates the pathogenic cell type being targeted in vivo. An ideal screening platform would utilize disease and organ-specific senescent cells as the screening target, coupled with a reporter system to validate whether the drug candidates are targeting the same cell type in vivo. However, a major challenge in the field has been isolating senescent cells in vivo for prospective studies, as early mouse reporters of senescence (based on the expression of *p16^Ink4a^*) have been mostly utilized to delete senescent cells for functional studies.

We recently generated an ultrasensitive fluorescent reporter of *p16^Ink4a^* (INKBRITE) that allowed us to identify cells with senescent characteristics in vivo as well as to isolate them from tissues (14). Utilizing this tool, we wanted to determine whether we could improve the existing senolytic screening pipeline by increasing the precision and scalability of HTS. We chose lung fibrosis as our model disease because *p16^Ink4a+^* cells have been previously reported to arise de novo in the fibrotic lung (19, 20, 26, 30), but the therapeutic role for existing senolytics is less clear. Utilizing our INKBRITE model, we identified specific *p16^Ink4a+^* cell types present in the fibrotic lungs of mice and humans, showing the contribution of senescent cells to a pathologic fibroblast subtype that was recently implicated in driving fibrotic remodeling (22, 23). More importantly, our single cell analysis demonstrated that the fate of *p16^Ink4a+^* fibroblasts is context dependent, as we did not observe the emergence of pathologic *p16^Ink4a+^* fibroblasts in a different injury model (naphthalene) with a different regenerative outcome. This supports the emerging view that senescent cells are not monolithic, but rather represent heterogeneous subpopulations that are capable of diverse responses to physiologic stimuli that can be harmful or beneficial (31). This is highlighted by our data that *p16^Ink4a+^* expression alone is not sufficient to drive the fibrotic response, but rather serves to prime the fibroblasts for maximal fibrotic induction in response to TGF-β1.

The context-dependent nature of *p16^Ink4a+^* fibroblasts would also suggest that their susceptibility to cell killing could differ across tissue states in vivo, thus providing the rationale for a senolytic platform that targets specific pathologic contexts. By using *p16^Ink4a+^* fibroblasts isolated from fibrotic lungs as the screening target while simultaneously using *p16^Ink4a–^* fibroblasts as the control within the same well, we were able to scale up the HTS to include over 2,000 compounds with known biologic activity. Furthermore, the ability to track these cells allowed us to utilize fibrotic INKBRITE lungs to validate the efficacy of candidate senolytics in deleting *p16^Ink4a+^* fibroblasts in preclinical models. This pipeline can be easily retrofitted to identify candidate senolytics in alternative disease models where *p16^Ink4a+^* cells play a pathogenic role. As part of our screening pipeline, we also leveraged the PCLS ex vivo culture system to validate our HTS and streamline our discovery platform. Our study highlights the potential utility of the PCLS for preliminary drug screening prior to in vivo experimentation. The capacity of PCLS to maintain fibrotic fibroblasts in their native tissue architecture and preserved extracellular environment, comparable to intact tissues, is critical (32). We showed high concordance in the efficacy of candidate senolytic in deleting *p16^Ink4a+^* cells when comparing PCLS ex vivo with whole animal in vivo. This approach can significantly streamline the screen and reduce the amount of animal testing, which is the most costly and time-consuming part of the pipeline. Further, it allows us to extend our studies into human diseased tissues in parallel with preclinical animal models to strengthen the target validation process (Supplemental Figure 8).

Our screening platform ultimately identified XL888, an HSP90 inhibitor, as the most promising senolytic in our screen for lung fibrosis. XL888 was one of the top hits in the initial HTS, and we were able to validate its potency in deleting *p16^Ink4a+^* fibroblasts in both a preclinical model of murine lung fibrosis as well as PCLS of IPF lung tissue. Importantly, XL888 attenuated multiple fibrotic indicators, including total collagen content, in the preclinical model of lung fibrosis. XL888 is currently in Phase I clinical trials as a potential anticancer agent in solid tumors (33), but our study would suggest a potential role in fibrotic diseases. While our screen was hypothesis-free, it was reassuring that we identified a compound class that has previously been shown to exhibit senolytic properties. HSP90 inhibitors such as geldamycin and 17-AAG were identified in a previous screen targeting progeroid fibroblasts in mice (29), although neither compound achieved our 3-σ threshold for secondary validation.

Together, our findings demonstrated that senescent cells, as identified by our INKBRITE reporter for *p16^Ink4a^*, contribute to profibrotic fibroblasts in pulmonary fibrosis. Furthermore, leveraging HTS with our in vivo reporter, we identified what we believe to be a new senolytic compound and validated its efficacy in fibrosis through direct measurement of senescent cell reduction in vivo along with attenuation of disease phenotype. We also described a method to isolate *p16^Ink4a+^* cells from human tissues to validate candidate senolytics in patient samples that will strengthen the rationale for clinical trials. A major caveat of our study is that clearly not all *p16^Ink4a+^* cells are functionally senescent. Our prior work had shown that senescent characteristics correlated with *p16^INK4a^* expression in the lung fibroblasts in vivo, which represented an analog expression of phenotypes rather than digital (on and off) property (14). This illustrates the complexity of senescent phenotypes in vivo that is not as well described compared with senescent cells in vitro, but also supports the necessity of screening against cellular targets in vivo to increase our understanding of how senolytics function to target specific subtypes of cells with senescent properties arising in vivo.

## Methods

### Sex as a biological variable.

Sex was not considered as a biological variable.

### Human lung samples.

Peripheral regions of the normal lungs were obtained to select for the distal regions of the lung from donors who in an irreversible coma and were rejected for lung transplantation. IPF lung specimens were taken from the periphery of the lung at the time of lung transplant. Age and sex of tissue donors are listed in Supplemental Table 3.

### Animal studies.

Mice between the ages of 8–12 weeks were used for the experiments with balance of gender between groups. C57BL/6J mice were obtained from Jackson Laboratory. Generation and genotyping of INKBRITE and *Dermo1^Cre/+^:p16^fl/fl^* lines were performed as previously described (14). For bleomycin-induced injury, mice were given pharmaceutical-grade bleomycin (Hospira) dissolved in PBS via intranasal instillation (2.5 U per kg body weight). For naphthalene injury, mice were administered with 300 mg/kg of naphthalene (Sigma-Aldrich) dissolved in corn oil by intraperitoneal injection. For XL888 treatment, mice were treated with 62.5 mg/kg via oral delivery 5 days a week for 2 weeks starting 10 days after bleomycin injury. XL888 was dissolved in 10 mM hydrochloric acid (HCL) with the concentration of 15.625 mg/mL. After vigorous vortexing, the dissolved XL888 was delivered to the mice using oral gavage daily. For DQ treatment in mice, C57BL/6J mice received a single dose of bleomycin (2.5 U/kg, day 1) and received dasatinib (5 mg/kg) and quercetin (50 mg/kg) at days 5, 11, and 17, following the previous study (21). Whole lung tissues from the mice were collected at day 23 for hydroxyproline assay.

### Histology and IHC.

For paraffin embedded mouse lungs, mouse right ventricles were perfused with 1 mL PBS and the lungs were inflated with 4% PFA, and then fixed in 4% PFA overnight at 4°C. After fixation, the lungs were washed by cold PBS 4 times in 2 hours at 4°C and dehydrated in a series of increasing ethanol concentration washes (30%, 50%, 70%, 95%, and 100%). The dehydrated lungs were incubated with Xylene for 1 hour at room temperature (RT) and with paraffin at 65°C for 90 minutes 2 times, and then embedded in paraffin and sectioned. Human and mouse PCLS samples were fixed in 4% PFA for 30 minutes. After PBS washes, slices were embedded in OCT after 30% sucrose incubations. Cryosections measuring 6–8 μm thick were used for IHC. The following antibodies were used: GFP (1:400, Abcam, ab6673), α smooth muscle actin (1:200, Abcam, ab5694), Transgelin (1:200, Abcam, ab14106), and Collagen I (1:200, Abcam, ab21286). Human lung fragments were fixed and processed as the mouse lungs. Antibodies used for human lung slide staining were ACTA2 (1:200, Abcam, ab5694), p16^INK4a^ (1:200, Santa Cruz, sc-56330), and CTHRC1 (1:200, Abcam, ab85739). Collagen staining was performed using Trichrome stain kit according to the manufacturer’s protocol (Abcam, ab150686). Images were captured using Zeiss Imager M1 or Leica Stellaris 5.

### RNA in situ.

Paraffin-embedded lung sections were used for RNA in situ detection of Cthrc1 using a RNAscope Multiplex Fluorescent Reagent kit (ACD biotechne) according to the manufacturer’s instructions.

### Lung digestion and fluorescence activated cell sorting.

Dissected mouse lung was tracheally perfused with a digestion cocktail of Collagenase Type I (225 U/mL, Thermo Fisher Scientific), Dispase (15 U/mL, Thermo Fisher Scientific) and Dnase (50 U/mL, Sigma-Aldrich) after perfusion with PBS and removed from the chest. The lung was incubated in a digestion cocktail for 45 minutes at 37°C with continuous shaking. The mixture was then washed with a FACS buffer (2% FBS and 1% Penicillin-Streptomycin in DMEM). The mixture was passed through a 70 μm cell strainer and resuspended in a red blood cell (RBC) lysis buffer, then passed through a 40 μm cell strainer. Cell suspensions were incubated with the appropriate conjugated antibodies in a sorting buffer for 30 minutes at 4°C and washed with FACS buffer. Doublets and dead cells were excluded based on forward and side scatter and SYTOX Blue (Invitrogen, S34857), respectively.

The following antibodies were used for staining: CD45-PE-Cy7 (Invitrogen, 50-112-9643), CD45-BV421 (BD, 563890), CD31-BV711 (BD, 740680), CD31-BV421 (Invitrogen, 48-0311-82), EpCAM-PE (BD, 563477), and EpCAM-BV421 (BD, 563214). Immune (CD45-biotin, Biolegend, 103104), epithelial (CD326-biotin, Biolegend, 118204), and endothelial (CD31-Biotin, Biolegend, 102404) cells were removed with EasySep mouse streptavidin RapidSpheres (StemCell, 19860A), when applicable. FACS was performed on a BD FACS Aria using FACSDiva Software. CD45^–^ CD31^–^ EpCAM^–^ cells were sorted for mesenchymal cells, the GFP^–^ and GFP^+^ fibroblasts were further separated and were sorted into FACS buffer. Analysis was performed using FlowJo software.

For the human lung, a distal piece (approximately 10 cm^3^) was dissected from the whole lung and washed with HBSS 4 times in 15 minutes. The piece of lung was further diced with razor blades and was added into the digestion cocktail of Collagenase Type I (225 U/mL, Thermo Fisher Scientific), Dispase (15 U/mL, Thermo Fisher Scientific) and Dnase (100 U/mL, Sigma-Aldrich). The mixture was incubated for 2 hours at 37°C and vortexed intermittently. The mixture was then liquefied with a blender and passed through 4×4 gauze, a 100 μm, and a 70 μm cell strainer. The mixture was resuspended in RBC lysis buffer, before passing through a 40 μm cell strainer. The cell suspensions were incubated with the antibodies in the FACS buffer for 30 minutes at 4°C and washed with the FACS buffer. The following antibodies were used for staining: CD45-APC-Cy7 (BioLegend, 304014), CD31-APC-Cy7 (BioLegend, 303120), CD11b-APC-Cy7 (BD Biosciences, 557754), and EpCAM-PE (BioLegend, 324206). DAPI (0.2 mg/mL) was used to exclude dead cells. Single cells were selected and CD45^–^ CD11b^–^ CD31^–^ EpCAM^–^ cells were sorted for mesenchymal cells. Cells were sorted into FACS buffer. FACS analysis was performed by FACSDiva (BD).

### qPCR.

Total RNA was obtained from cells using PicoPure RNA Isolation Kit (Applied Biosystems, KIT0204) or RNeasy mini kit (QIAGEN, 74106), following the manufacturers’ protocols. cDNA was synthesized from total RNA using the SuperScript Strand Synthesis System (Thermo Fisher Scientific, 18080044). Quantitative reverse transcription–PCR (qRT-PCR) was performed using the SYBR Green system (Thermo Fisher Scientific, F415L). Relative gene expression levels after qRT-PCR were defined using the ΔΔCt method and normalizing to the housekeeping genes. The qRT-PCR primers used for mouse are as follows: Cthrc1-F: CAGTTGTCCGCACCGATCA; Cthrc1-R: GGTCCTTGTAGACACATTCCATT; Col1a1-F; TGACTGGAAGAGCGGAGAGT; Col1a1-R:GTTCGGGCTGATGTACCAGT; Col3a1-F: CTGTAACATGGAAACTGGGGAAA; Col3a1-R: CCATAGCTGAACTGAAAACCACC; Spp1-F: AGCAAGAAACTCTTCCAAGCAA; Spp1-R: GTGAGATTCGTCAGATTCATCCG; Acta2-F: ACTCTCTTCCAGCCATCTTTCA; Acta2-R: ATAGGTGGTTTCGTGGATGC; Postn-F: TGGTATCAAGGTGCTATCTGCG; Postn-R: AATGCCCAGCGTGCCATAA; S100a4-F: TGAGCAACTTGGACAGCAACA; S100a4-R: CTTCTTCCGGGGCTCCTTATC; Tagln-F: GGTGGCTCAATTCTTGAAGGC; Tagln-R: TGCTCCTGGGCTTTCTTCATA; p16^INK4a^-F: AATCTCCGCGAGGAAAGC; p16^INK4a^-R: GTCTGCAGCGGACTCCAT; p21-F: TAAGGACGTCCCACTTTGCC; p21-R: CGTCTCCGTGACGAAGTCAA; Gapdh-F: GGCCCCTCCTGTTATTATGGGGGT; and Gapdh-R: CCCCAGCAAGGACACTGAGCAAGA. The primers used for human are as follows: *p16^INK4a^*-F: GTCGGGTAGAGGAGGTGCG; *p16INK4a*-R: CATGACCTGGATCGGCCTC; *p21*-F: TTGTACCCTTGTGCCTCGCT; *p21*-R: CGTTTGGAGTGGTAGAAATCTGTC; *CTHRC1*-F: GTGGCTCACTTCGGCTAAAAT; *CTHRC1*-R: CACTAATCCAGCACCAATTCCTT; *HAS1*-F: TCAAGGCGCTCGGAGATTC; *HAS1*-R: CTACCCAGTATCGCAGGCT; *SPP1*-F: GAAGTTTCGCAGACCTGACAT; *SPP1*-R: GTATGCACCATTCAACTCCTCG; *PostN*-F: CTCATAGTCGTATCAGGGGTCG; *PostN*-R: ACACAGTCGTTTTCTGTCCAC; *ACTA2*-F: AAAAGACAGCTACGTGGGTGA; *ACTA2*-R: GCCATGTTCTATCGGGTACTTC; *COL1A1*-F: GGGGTAAGTCCCTTTCTGCC; *COL1A1*-R: ATTGCCTTTGATTGCTGGGC; and *RPL19*-F: CCCATCTTTGATGAGCTTCC; *RPL19*-R: TGCTCAGGCTTCAGAAGAGG.

### Single-cell RNA sequencing and analysis.

Single cell sequencing was performed on a 10× Chromium instrument (10× Genomics) at the Institute of Human Genetics (UCSF, San Francisco, California, US). Briefly, live mouse lung cells were sorted and resuspended in 50 μL PBS with 0.04% BSA at 1,000 cells/μL and loaded onto a single lane into the Chromium Controller to produce gel bead-in emulsions (GEMs). GEMs underwent reverse transcription for RNA barcoding and cDNA amplification. The library was prepped with the Chromium Single Cell 3′ Reagent Version 3 kit. The samples were sequenced using the HiSeq2500 (Illumina) in Rapid Run Mode. We used the Seurat R package along with a gene-barcode matrix provided by CellRanger for downstream analysis. Following the standard workflow of Seurat, we generated Seurat objects after using ScaleData, RunPCA, and RunUMAP. For human scRNA-Seq data, we used processed scRNA-Seq data from normal and IPF lungs from GSE147066. After generating subsets of lung fibroblasts, violin plots and density plots were generated.

### Cell culture.

Freshly isolated mesenchymal cells from INKBRITE lungs (GFP^–^ or GFP^+^) or human lung fibroblasts were cultured in DMEM/F-12 (Thermo Fisher Scientific, 11330032) with 10% FBS and 1% Penicilin/Streptomycin. The medium was changed every 2 days and lung fibroblasts were maintained for no more than 3 passages.

### TGF-β1 in vitro stimulation.

Fibroblasts were sorted from fibrotic INKBRITE lungs. 1 × 10^5^ cells were seeded into 48-well plates and cultured in DMEM/F12 with 2% FBS and 1% Penicillin/Streptomycin for 24 hours. Medium was changed to serum free DMEM with 1% Penicillin/Streptomycin for 24 hours. After the serum starvation, medium was changed to serum-free DMEM/F-12 with 1% Penicillin/Streptomycin and 1 ng/mL TGF-β1 (Peprotech, 10778-032). After 24–48 hours of stimulation, RNA was obtained using RNeasy mini kit (QIAGEN, 74106).

### Lentivirus infection.

Primary human lung fibroblasts were seeded and infected the following day with lentivirus (Lenti-tTS/rtTA, Lenti-TRE-p16INK4a-T2A-dTomato). On day 1, the fibroblasts were infected with lentivirus at 5 multiplicity of infection (MOI) in DMEM-F12 with 10% FBS and polybrene at 5 μg/mL. On day 2, cells were washed with 1 × PBS 4 times and placed on regular media (DMEM-F12, 10%FBS, 1% PS). Doxycycline (1 μg/mL) treatment began 72–96 hours later for Lenti-tTS/rtTA and Lenti-TRE-p16 dual–transduced cells.

### Adenovirus infection.

Primary mouse lung fibroblasts were seeded and infected the following day with adenovirus expressing GFP. On day 1, the fibroblasts were infected with adenovirus at 260 MOI in DMEM-F12 with 10% FBS. On day 2, cells were washed with 1 × PBS 4 times and placed on regular media (DMEM-F12, 10%FBS, 1% Penicillin/Streptomycin). Cells were treated with XL888 from day 3 to day 6.

### HTS.

The screen was performed in collaboration with the Small Molecule Discovery Center (SMDC) at UCSF. INKBRITE mice received a single dose of bleomycin at 2.5 U/kg and lung tissues were collected 14 days after the injury. Single cell suspension of the lung was prepared and sorted as described above. A suspension of GFP^+^ and GFP^–^ fibroblast cells (1:1 ratio) was plated in 384-well plates (Greiner Bio-1, 781096) at 2,000 cells/well density using WellMate liquid dispenser (Matrix). After an overnight incubation, 2,400 test compounds from SelleckChem bioactive and epigenetic library were added using Biomek FXp (Beckman Coulter) pin tools to a final concentration of 1 μM, followed by a 3-day incubation. On the third day, plates were washed with 1 × PBS and NucView530 dye mix (Biotium) was dispensed at 2 μM final concentration into each well using EL406 washer dispenser (BioTek), followed by an hour incubation. Plates were then washed with 1 × PBS and fixed with an addition of 4% PFA + 1 μg/mL Hoechst mix into each well. After 15 minutes’ incubation, plates were washed with 1 × PBS and imaged. Fluorescence images were captured using IN Cell Analyzer 6500HS (Cytiva) at 20 × magnification, 4 field-of-views per well. Images were processed using IN Cell Developer Tool Box. Nuclear mask was created from the Hoechst channel and was applied to both GFP and NucView channels to calculate the number of total cells, GFP^+^, GFP^–^, and NucView^+^ cells.

Compounds that caused GFP^+^ cell count to be below the 3SD limit of the negative control (wells with no test compound) were determined as hit candidates. Thirty-two compounds were selected for a follow up validation screen based on compounds’ identity and targets. Dose response curves and their IC_50_s were generated for these 32 compounds with the concentration ranging between 0.005 μM to 2.5 μM (2-fold dilution).

### Generation of PCLS culture.

For mouse PCLS, INKBRITE mice were injured with 2.5 U/kg of bleomycin and lung tissues were collected 2 weeks after the injury. The lungs were perfused with PBS through the right ventricle and inflated with 1–2 mL of 2% agarose (Thermo Fisher Scientific, 16550100) dissolved in PBS by trachea. After inflation, the trachea was tied with a suture to prevent agarose leakage. Lungs were dissected from the chest cavity and submerged in ice-cold PBS to solidify agarose. Lung lobes were sliced at a width of 500 μm using a vibratome (Leica, VT 1000S).

For human PCLS, fresh lung tissues were obtained from IPF patients that underwent lung transplantation. After washing with PBS, the tissues were inflated with warm 2% agarose and placed in cold PBS. The lung specimens were cut into strips and sliced into 600 μm–thick slices using a vibratome.

The slices were cultured in DMEM/F-12 (Thermo Fisher Scientific, 11330032) with 1% Penicillin/Streptomycin under standard cell culture conditions (37C, 5% CO_2_). ABT263 (2.5 μM), ABT737 (2 μM), Fisetin (10 μM), DQ (1 μM + 20 μM), and XL888 (1 μM) were treated during the culture. A concentration of 1 μM was used for other candidate compounds (TSA, ganetespib, fimepinostat, and dacinostat). At day 5, cultured PCLSs were processed for downstream analyses.

### Flow cytometry analysis of mouse PCLS.

The lung slices were placed into 15 mL conical tubes containing 1 mL of digestion cocktail of Dispase (3 U/mL, Thermo Fisher Scientific) and Dnase (50 U/mL, Sigma-Aldrich) after PBS washes. The slices were incubated in a digestion cocktail for 30 minutes at 37°C with continuous shaking. The mixture was then washed with a FACS buffer (2% FBS and 1% Penicillin-Streptomycin in DMEM). The mixture was passed through a 70 μm cell strainer. Cells were stained with antibodies and analyzed by flow cytometry as described above.

### Hydroxyproline assay.

Collagen content in the lungs was assessed by measuring the hydroxyproline level using the Hydroxyproline Colorimetric Assay Kit (K555-100) from BioVision. Briefly, lung tissue was homogenized in water and the homogenized samples were hydrolyzed by incubation with 12N hydrochloric acid at 120°C for 3 hours. The hydrolysates were oxidized using chloramine T, followed by incubation with Ehrlich’s perchloric acid reagent. Absorbance was measured at 560 nm.

### CT^FR^.

Isolated fibroblasts were cultured for 3 days and stained with CT^FR^ reagent. The fibroblasts were detached and stained with 1 μM of CellTrace for 20 minutes at 37°C (1 million cells/mL) following the manufacturer’s protocol. After staining, the cells were washed with media and cultured for 3–4 days. Serum-starved cells after CT^FR^ staining were used to separate CT^FR-hi^ and CT^FR-lo^ cells based on CT^FR^ levels. CT^FR^ stained cells within the high intensity range of 95% to 97% encompassing serum-starved, nonproliferating cells were sorted as CT^FR-hi^ cells, while cells exhibiting a lower intensity range were sorted as CT^FR-lo^ cells.

### Statistics.

All data are presented as mean ± SD. Statistical differences between the groups were compared using unpaired 2-tailed Student’s *t* test or 1-tailed Student’s *t* test for 2 groups or 1-way ANOVA for multiple groups. Statistical significance was defined as *P < 0.05, **P < 0.01, ***P < 0.001. Statistical details and the number of replicates for each experiment can be found in the figure legends. The following formula is used to compute sample size and power: 

 Equation 1


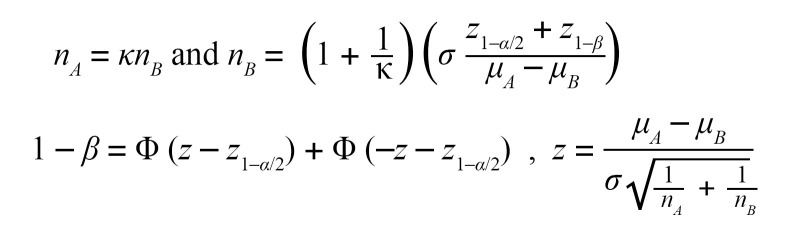


where κ = *nA*/*nB*κ = nA/nB is the matching ratio; σ is standard deviation; Φ is the standard normal distribution function; Φ−1 is the standard normal quantile function; α is Type I error; and β is Type II error, meaning 1−β is power.

### Study approval.

Animal husbandry and all experiments were conducted under IACUC-approved protocols at University of California, San Francisco (no. AN191522-01J). Human specimen isolation from explanted lungs of patients undergoing lung transplantation at UCSF is approved by Institutional Review Board at UCSF (no. 13-10738). All participants provided written informed consent.

### Data availability.

Previously published human scRNA-Seq data that were reanalyzed in this study are available in NCBI Gene Expression Omnibus (GEO) under the accession number GSE147066. The sequencing data of the mouse that support the findings of this study have been deposited in the accession number GSE235352. The values for all data points in graphs are reported in the Supporting Data Values file.

## Author contributions

JYL and TP conceived the experiments and wrote the manuscript. JYL, SR, MZ, MK, NSR, CR, GP, CW, KKHA, and TT performed the experiments. PJW provided clinical specimen. DS and MRA provided expertise and feedback.

## Supplementary Material

Supplemental data

Supplemental table 1

Supplemental table 2

Supplemental table 3

Supporting data values

## Version 1 03/07/2024

In-Press Preview

## Version 2 05/01/2024

Electronic publication
