# Supplementary material for: An in vivo screening platform identifies senolytic compounds that target p16INK4a+ fibroblasts in lung fibrosis
Source: J Clin Invest. 2024 Mar 7;134(9):e173371. doi: 10.1172/JCI173371 (PMC11060735; doi:10.1172/JCI173371)
Supplement: Supplemental data [file jci-134-173371-s147.pdf]

Supplementary Figure Legends

Supplementary Figure 1. Comparison of *p16*^{Ink4a}+ fibroblasts arising in non-fibrotic airway injury (naphthalene) vs. fibrotic alveolar injury (bleomycin).

(A) Uniform Manifold Approximation and Projection (UMAP) of single cell RNA sequencing data of *p16*^{Ink4a}+ fibroblasts from uninjured, naphthalene injured (14 dpi), and bleomycin (14 dpi) injured lungs.

(B) Proportion of *p16*^{Ink4a}+ fibroblasts categorized within different fibroblast subtypes in uninjured, naphthalene, and bleomycin injured lungs.

(C) Uniform Manifold Approximation and Projection (UMAP) of single cell RNA sequencing data of *Col1a1*+ fibroblasts from uninjured and bleomycin (14 dpi) injured lungs.

(D) Proportion of *Col1a1*+ fibroblasts categorized within different fibroblast subtypes in uninjured and bleomycin injured lungs.

(E) Feature plots of genes enriched in the pathologic fibroblast subset arising in bleomycin injury.

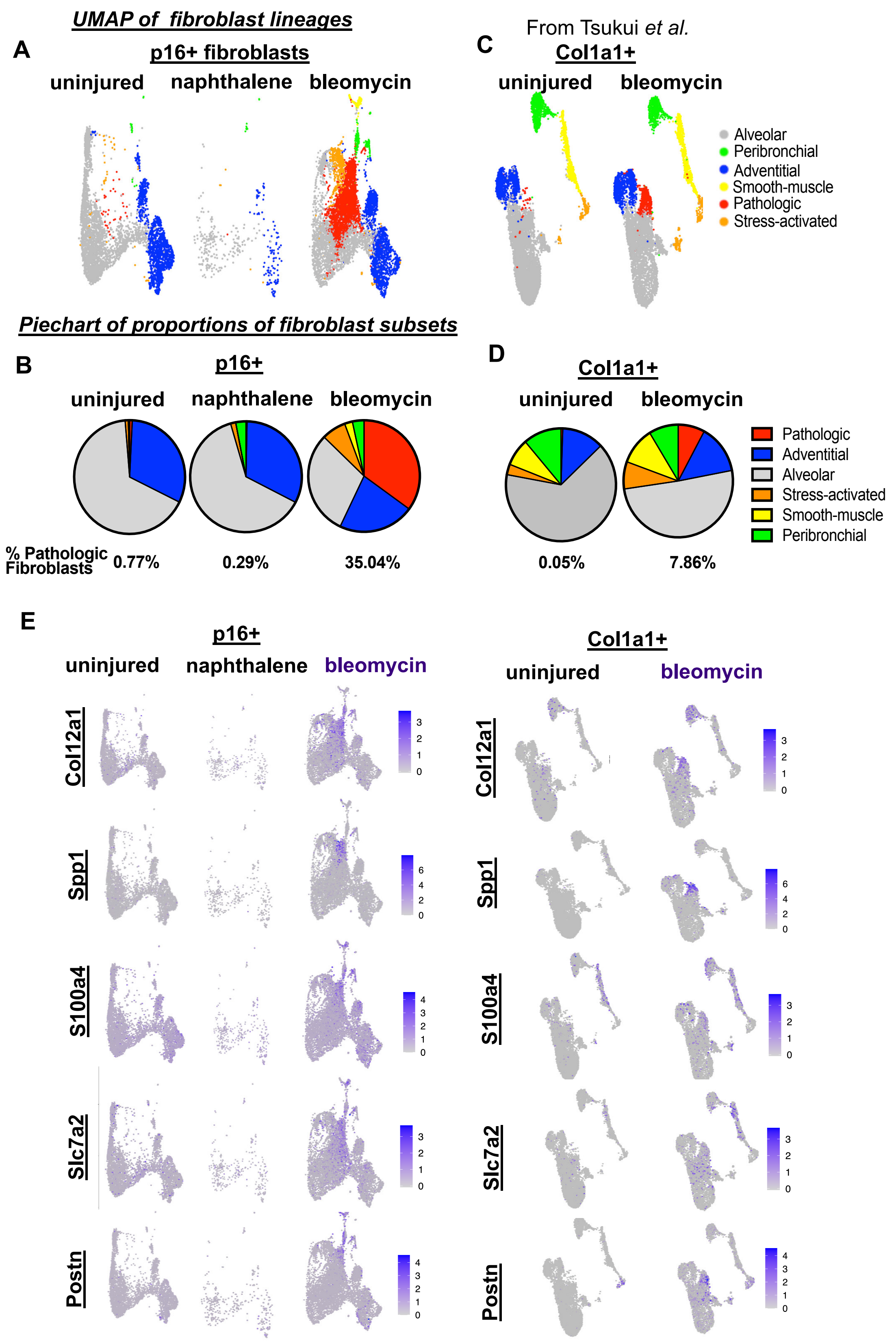
UMAP of fibroblast lineages
A
p16+ fibroblasts
uninjured
naphthalene
bleomycin
C
From Tsukui et al.
Col1a1+
uninjured
bleomycin
Alveolar
Peribronchial
Adventitial
Smooth-muscle
Pathologic
Stress-activated
Piechart of proportions of fibroblast subsets
B
p16+
uninjured
naphthalene
bleomycin
% Pathologic Fibroblasts
0.77%
0.29%
35.04%
D
Col1a1+
uninjured
bleomycin
Pathologic
Adventitial
Alveolar
Stress-activated
Smooth-muscle
Peribronchial
0.05%
7.86%
E
p16+
uninjured
naphthalene
bleomycin
Col1a1+
uninjured
bleomycin
Col12a1
Spp1
S100a4
Slc7a2
Postn

Supplementary Figure 2. Characteristics of *p16^Ink4a^*+ fibroblasts isolated from fibrotic INKBRITE lungs.

(A) qPCR of $p16^{Ink4a}$ and *p21* expressions in GFP- and GFP+ fibroblasts from fibrotic INKBRITE lungs (n = 6 biological replicates, experiment repeated 2X).

(B) Immunofluorescence analysis and quantification of γH2AX of $p16^{Ink4a}$- and $p16^{Ink4a}$+ fibroblasts from fibrotic INKBRITE lungs (n = 15 technical replicates, experiment repeated 2X). Scale bars, 100 μm.

(C) SA-β-gal staining and quantification of $p16^{Ink4a}$- and p $p16^{Ink4a}$+ (n = 3 technical replicates, experiment repeated 2X). Scale bars, 200 μm.

(D) Comparison of cellular size of freshly isolated $p16^{Ink4a}$- and $p16^{Ink4a}$+ fibroblasts (n = 12 biological replicates).

(E) Comparison of non-proliferating cell's percentage by CellTrace labeling reagent (n = 3 technical replicates, experiment repeated 2X).

Data are represented as mean ± SD.; *$P < 0.05$; **$P < 0.01$; ***$P < 0.001$; two-tailed Student's t test (A-E).

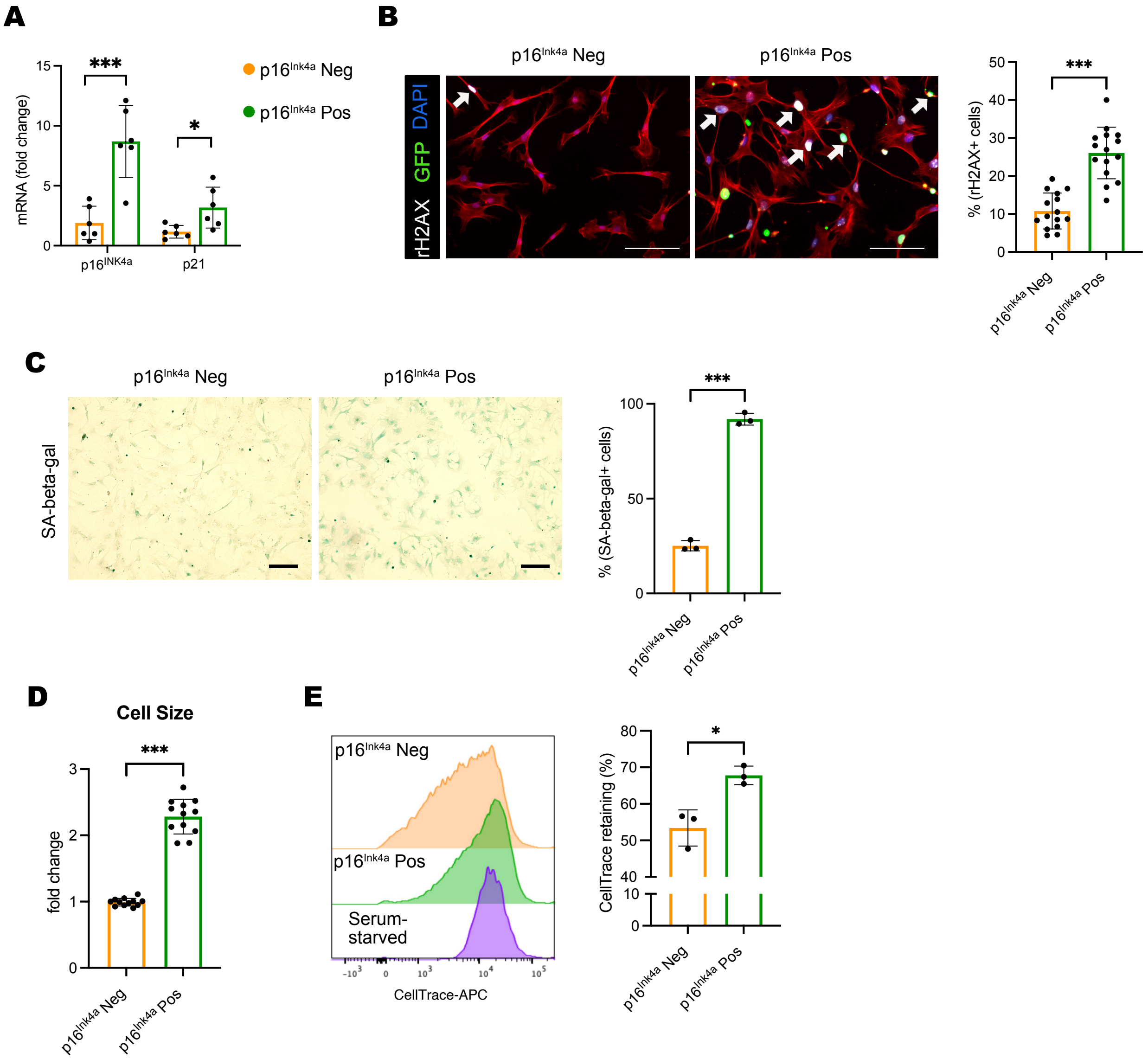
A
B
C
D
E
mRNA (fold change)
p16INK4a
p21
p16Ink4a Neg
p16Ink4a Pos
rH2AX GFP DAPI
% (rH2AX+ cells)
SA-beta-gal
% (SA-beta-gal+ cells)
Cell Size
fold change
Serum-starved
CellTrace-APC
CellTrace retaining (%)

Supplementary Figure 3. Comparison of pathologic markers in *p16^{Ink4a}*+ vs. *p16^{Ink4a}*- fibroblasts isolated from fibrotic INKBRITE lungs.

(A) Immunofluorescence analysis and quantification of ACTA2+ fibroblasts on cytospin of $p16^{Ink4a}$- and $p16^{Ink4a}$+ fibroblasts isolated from fibrotic INKBRITE lungs (n = 7 biological replicates, experiment repeated 2X).

(B) Immunofluorescence analysis and quantification of COL1+ fibroblasts on cytospin of $p16^{Ink4a}$- and $p16^{Ink4a}$+ fibroblasts isolated from fibrotic INKBRITE lungs (n = 7 biological replicates, experiment repeated 2X).

(C) Immunofluorescence analysis and quantification of CTHRC1+ fibroblasts on cytospin of $p16^{Ink4a}$- and $p16^{Ink4a}$+ fibroblasts isolated from fibrotic INKBRITE lungs (n = 7 biological replicates, experiment repeated 2X).

Data are represented as mean ± SD.; *$P < 0.05$; **$P < 0.01$; ***$P < 0.001$; two-tailed Student's t test (A-C). Scale bars, 50 μm.

A

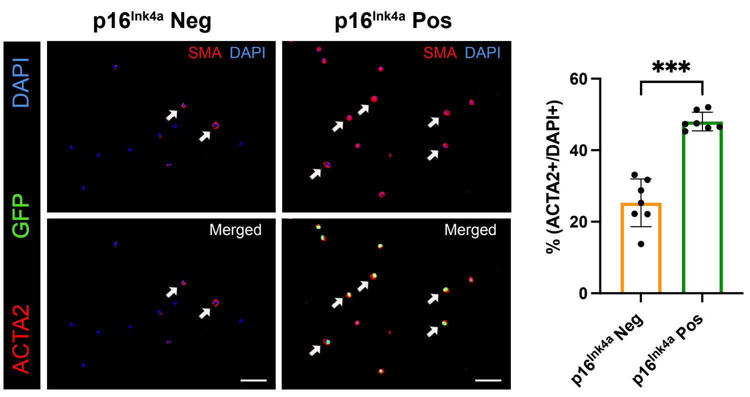
p16Ink4a Neg
p16Ink4a Pos
SMA DAPI
Merged
DAPI
GFP
ACTA2
***
% (ACTA2+/DAPI+)
60
40
20
0
p16Ink4a Neg
p16Ink4a Pos

B

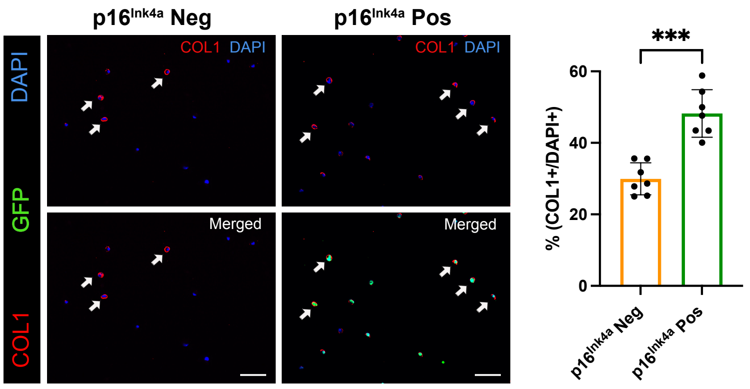
p16Ink4a Neg
p16Ink4a Pos
COL1 DAPI
Merged
DAPI
GFP
COL1
***
% (COL1+/DAPI+)
60
40
20
0
p16Ink4a Neg
p16Ink4a Pos

C

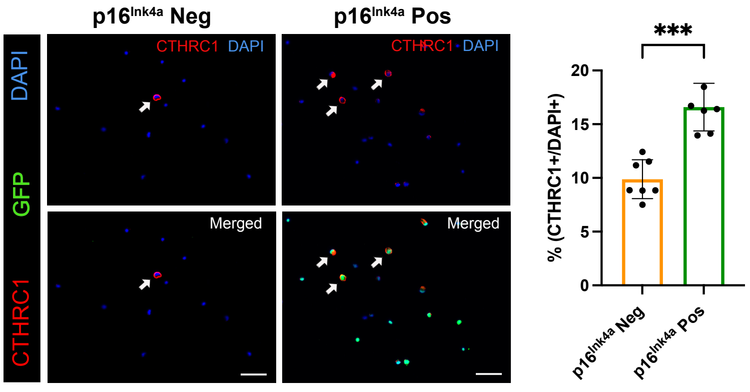
p16Ink4a Neg
p16Ink4a Pos
CTHRC1 DAPI
Merged
DAPI
GFP
CTHRC1
***
% (CTHRC1+/DAPI+)
20
15
10
5
0
p16Ink4a Neg
p16Ink4a Pos

Supplementary Figure 4. Dose response curves of *p16^{Ink4a}*+ vs. *p16^{Ink4a}*- fibroblasts isolated from fibrotic lungs when treated with top candidates from primary screen, along with calculation of selectivity index (based on IC50) and efficacy ratio (based on E_{max}).

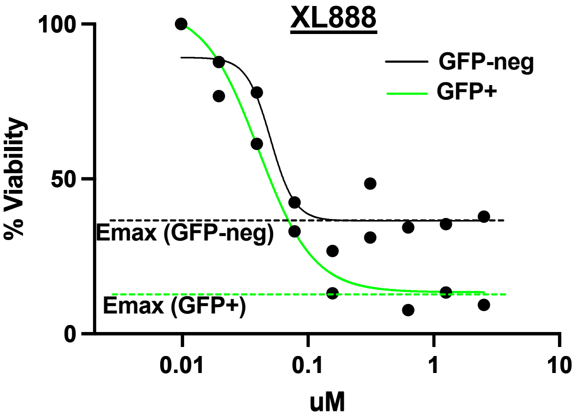

Selectivity Index (SI) = IC50 GFP-neg viability/ IC50 GFP+ viability = 1.3

Efficacy Ratio (ER) = Emax GFP-neg viability/ Emax GFP+ viability = 4.1

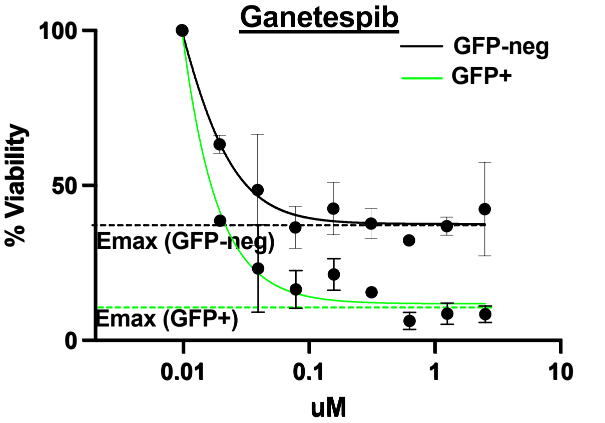

Selectivity Index (SI) = 47.4

Efficacy Ratio (ER) = 4.9

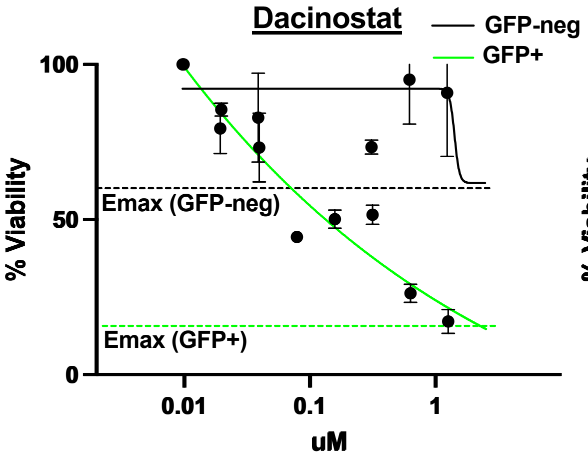

Selectivity Index (SI) = 1.5E10

Efficacy Ratio (ER) = 4.1

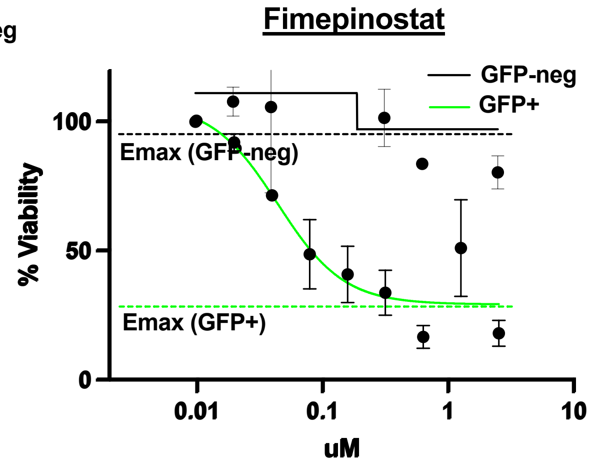

Selectivity Index (SI) = 7.1

Efficacy Ratio (ER) = 4.6

Supplementary Figure 5. Analysis of precision cut lung slices (PCLS) from fibrotic INBRITE animals cultured with senolytic compounds.

(A) Viability of PCLS cultured after 5 days (n = 52 technical replicates).

(B, C) Percentages of GFP+ fibroblasts (%GFP of all fibroblasts) from PCLS after culture with indicated compounds (n = 4 technical replicates, experiment repeated 2X).

(D) Percentages of GFP+ fibroblasts (%GFP of all fibroblasts) from PCLS after culture with published senolytics and XL888 (n = 4 technical replicates, experiment repeated 2X).

(E) Effect of XL888 on viability of GFP expressing vs. nonexpressing lung fibroblasts.

(F) (Top) Percentages of GFP+ fibroblasts (%GFP of all fibroblasts) from PCLS isolated from naphthalene injured INKBRITE after culture with XL888 (n = 5 technical replicates). (Bottom) Immunofluorescence analysis of ACTA2, COL1A1, and GFP in mouse PCLS treated with vehicle or XL888. Scale bars, 50 μm.

Data are represented as mean ± SD.; **P* < 0.05; ***P* < 0.01; ****P* < 0.001; one-way ANOVA (B-D); or two-tailed Student's t test (F).

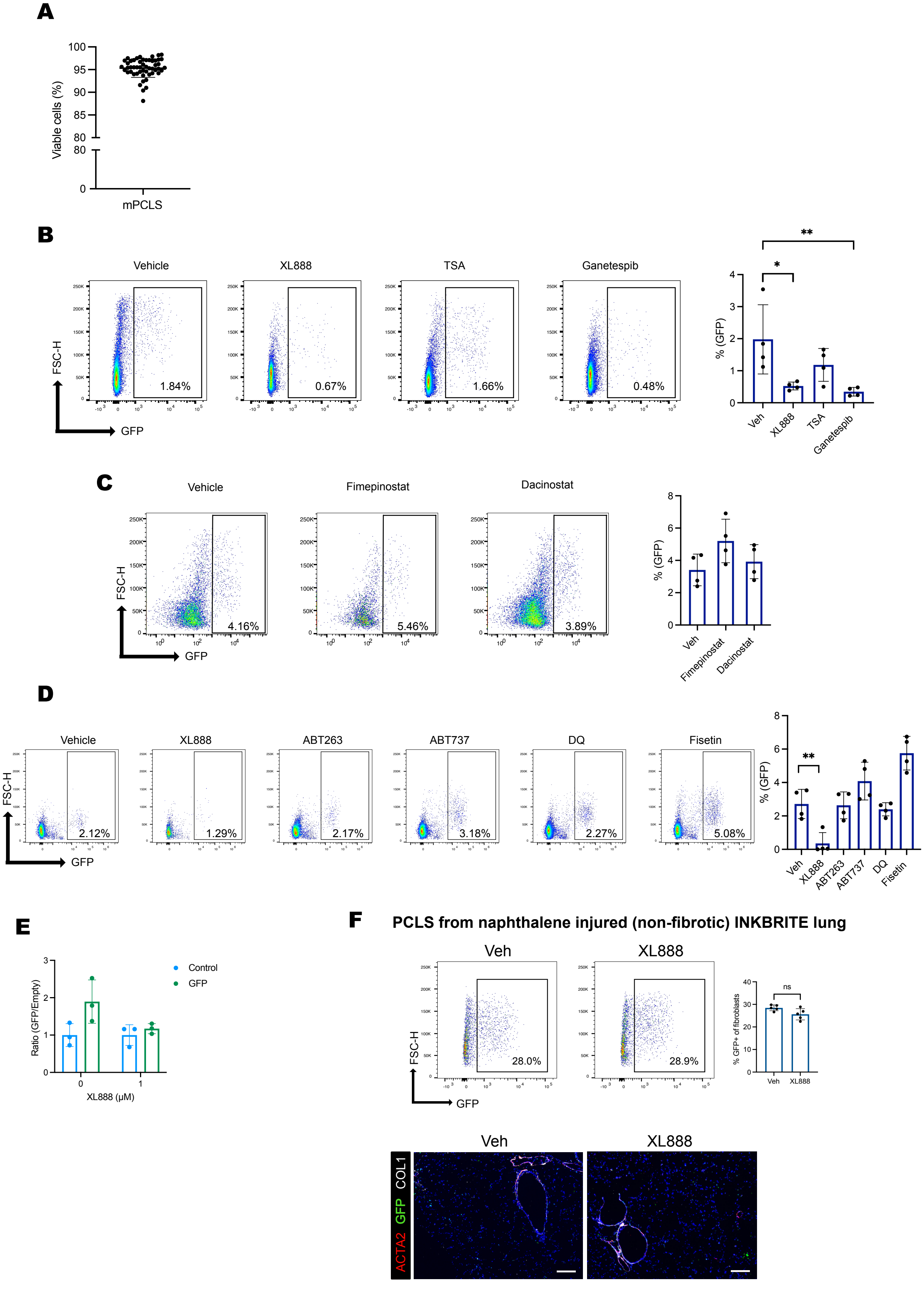
A
Viable cells (%)
mPCLS
B
Vehicle
XL888
TSA
Ganetespib
1.84%
0.67%
1.66%
0.48%
FSC-H
GFP
% (GFP)
Veh
XL888
TSA
Ganetespib
C
Vehicle
Fimepinostat
Dacinostat
4.16%
5.46%
3.89%
D
Vehicle
XL888
ABT263
ABT737
DQ
Fisetin
2.12%
1.29%
2.17%
3.18%
2.27%
5.08%
E
Control
GFP
Ratio (GFP/Empty)
XL888 (μM)
F
PCLS from naphthalene injured (non-fibrotic) INKBRITE lung
Veh
XL888
28.0%
28.9%
% GFP+ of fibroblasts
ns
ACTA2 GFP COL1

Supplemental Figure 6. Analysis of selected senolytics administered *in vivo*

(A) Flow cytometry analysis of GFP+ fibroblasts (%GFP of all fibroblasts) from INKBRITE lungs after administration of vehicle or Ganetespib (n = 13-14 biological replicates).

(B) Flow cytometry analysis of GFP+ fibroblasts from INKBRITE lungs after administration of vehicle or Trichostatin A (TSA) (n = 11-13 biological replicates).

(C) Flow cytometry analysis of GFP+ fibroblasts from INKBRITE lungs after administration of vehicle or Fimepinostat (n = 6-8 biological replicates).

(D) Flow cytometry analysis of GFP+ fibroblasts from INKBRITE lungs after administration of vehicle or Dacinostat (n = 2-10 biological replicates).

(E) Flow cytometry analysis of GFP+ fibroblasts from fibrotic INKBRITE lungs after administration of vehicle or dasatinib and quercetin (DQ) (n = 7-8 biological replicates).

(F) Flow cytometry analysis of GFP+ lineages from fibrotic INKBRITE lungs after administration of vehicle or dasatinib and quercetin (DQ) (n = 7-8 biological replicates).

(G) qPCR analysis of fibrotic genes expression from whole lung of vehicle or DQ treated fibrotic lungs (n = 8 biological replicates).

(H) Quantitative analysis of collagen of bleomycin-injured lungs after vehicle or DQ administration (n = 5-7 biological replicates).

Data are represented as mean ± SD.; *$P < 0.05$; **$P < 0.01$; ***$P < 0.001$; two-tailed Student's t test (A-H).

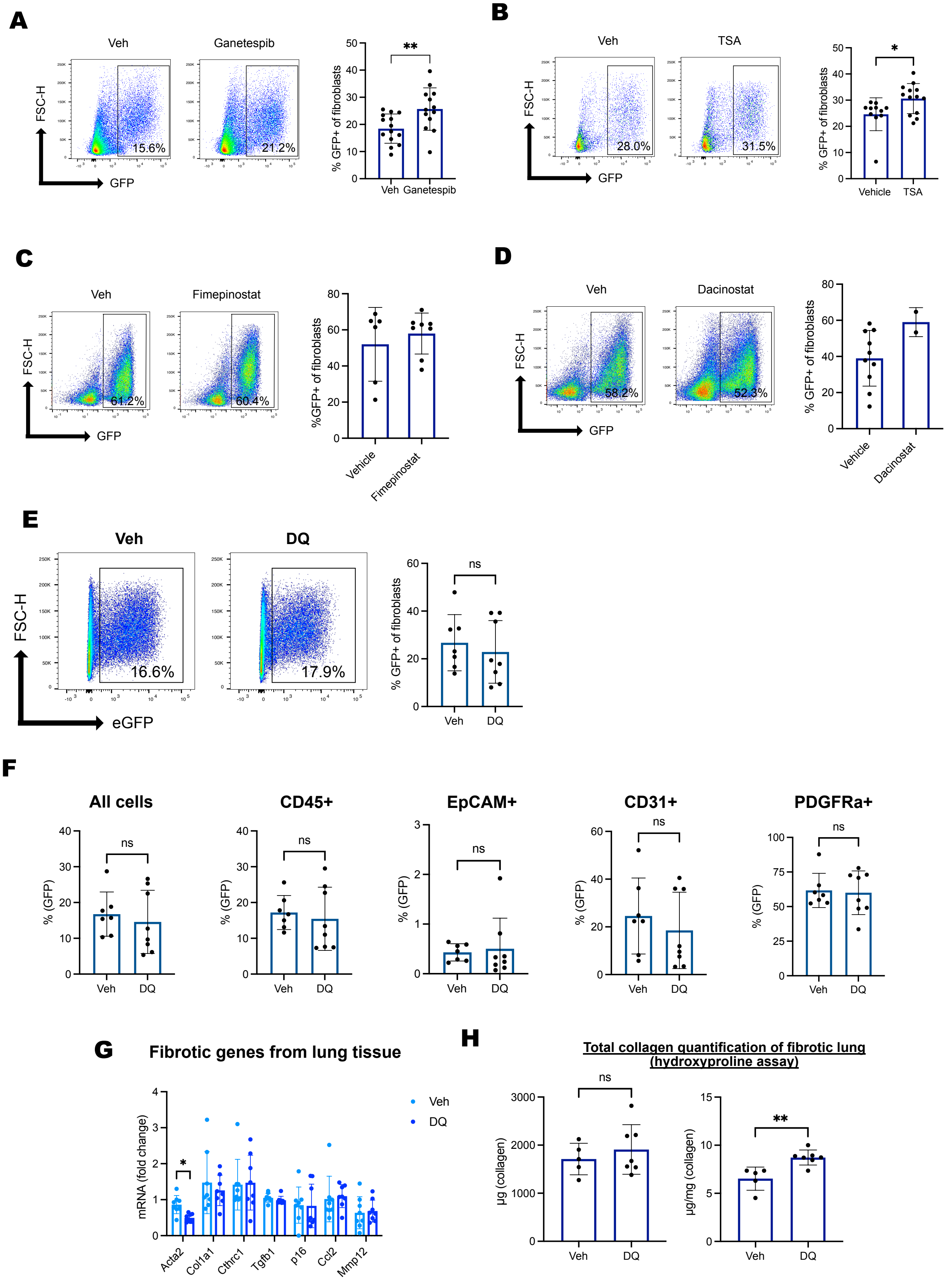
A
Veh
Ganetespib
FSC-H
GFP
15.6%
21.2%
% GFP+ of fibroblasts
**
Veh
Ganetespib
B
Veh
TSA
28.0%
31.5%
*
Vehicle
TSA
C
Veh
Fimepinostat
61.2%
60.4%
%GFP+ of fibroblasts
Vehicle
Fimepinostat
D
Veh
Dacinostat
58.2%
52.3%
Vehicle
Dacinostat
E
Veh
DQ
eGFP
16.6%
17.9%
ns
F
All cells
CD45+
EpCAM+
CD31+
PDGFRa+
% (GFP)
G
Fibrotic genes from lung tissue
mRNA (fold change)
Acta2
Col1a1
Cthrc1
Tgfb1
p16
Ccl2
Mmp12
H
Total collagen quantification of fibrotic lung (hydroxyproline assay)
µg (collagen)
µg/mg (collagen)

Supplemental Figure 7. Analysis of *p16^{INK4a}*+ fibroblasts in human lungs with IPF.

(A) Dot plot showing representative markers for each fibroblast cluster of human lung scRNA-seq data from IPF and control patients.

(B) qPCR analysis of senescence and fibrotic markers in control and IPF fibroblasts (n = 3 biological replicates).

(C) Immunofluorescence analysis of CTHRC1 and p16^{INK4a} in IPF lung section. Scale bar, 100 μm.

(D) qPCR analysis of *p14^{ARF}* expression in *p16^{INK4a}* high and low fibroblasts isolated from patients with IPF (n = 5 biological replicates).

(E) Viability of human IPF PCLS cultured after 5 days (n = 18 technical replicates).

Data are represented as mean ± SD.; *$P < 0.05$; **$P < 0.01$; ***$P < 0.001$; two-tailed Student's t test (B-D).

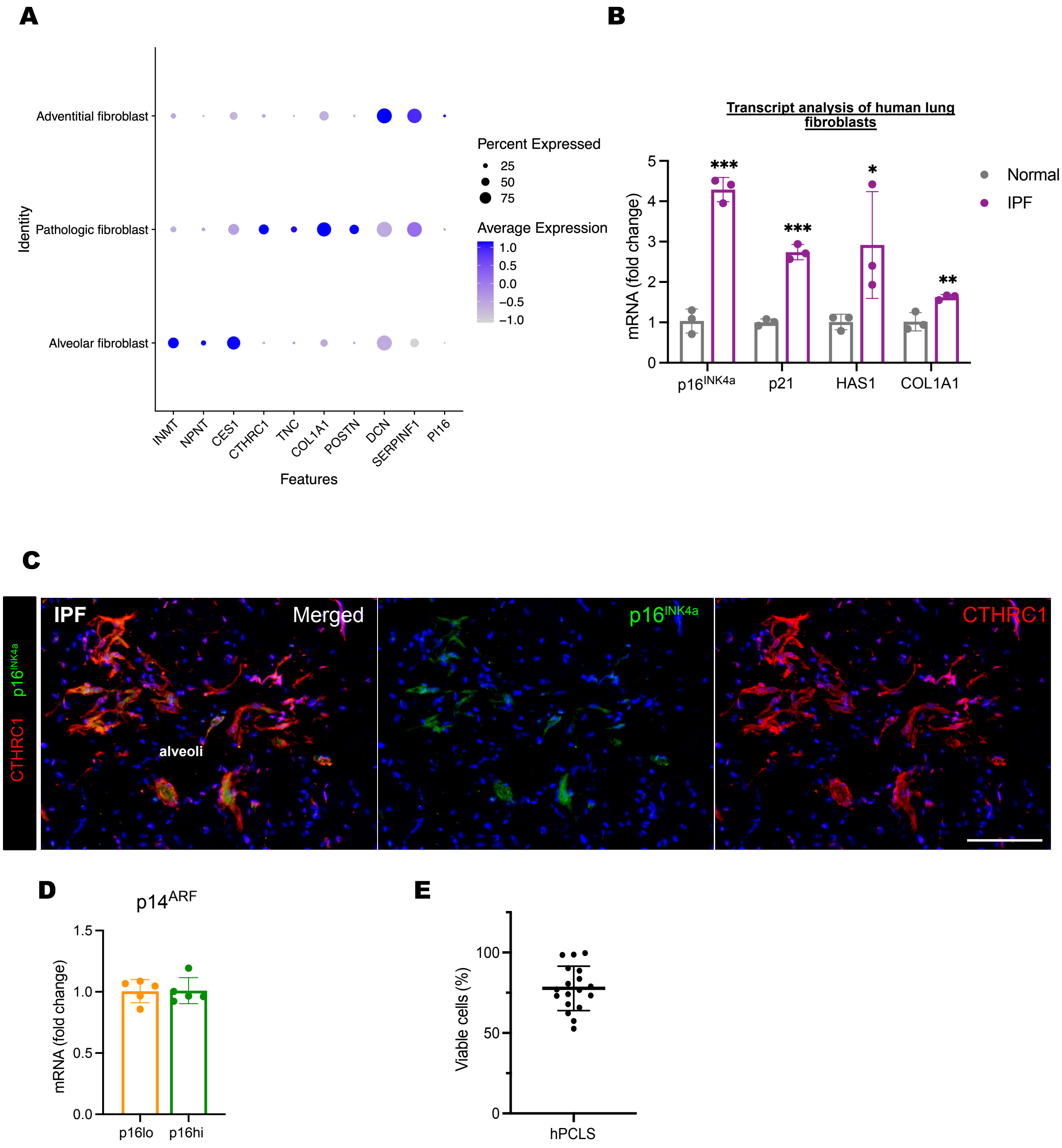
A
Adventitial fibroblast
Pathologic fibroblast
Alveolar fibroblast
Identity
INMT
NPNT
CES1
CTHRC1
TNC
COL1A1
POSTN
DCN
SERPINF1
PI16
Features
Percent Expressed
25
50
75
Average Expression
1.0
0.5
0.0
−0.5
−1.0
B
Transcript analysis of human lung fibroblasts
mRNA (fold change)
0
1
2
3
4
5
***
***
*
**
p16INK4a
p21
HAS1
COL1A1
Normal
IPF
C
IPF
Merged
alveoli
p16INK4a
CTHRC1
D
p14ARF
mRNA (fold change)
0.0
0.5
1.0
1.5
p16lo
p16hi
E
Viable cells (%)
0
50
100
hPCLS

Supplemental Figure 8. Overview of screening platform targeting *$p16^{INK4a}$*+ fibroblasts *in vivo*.

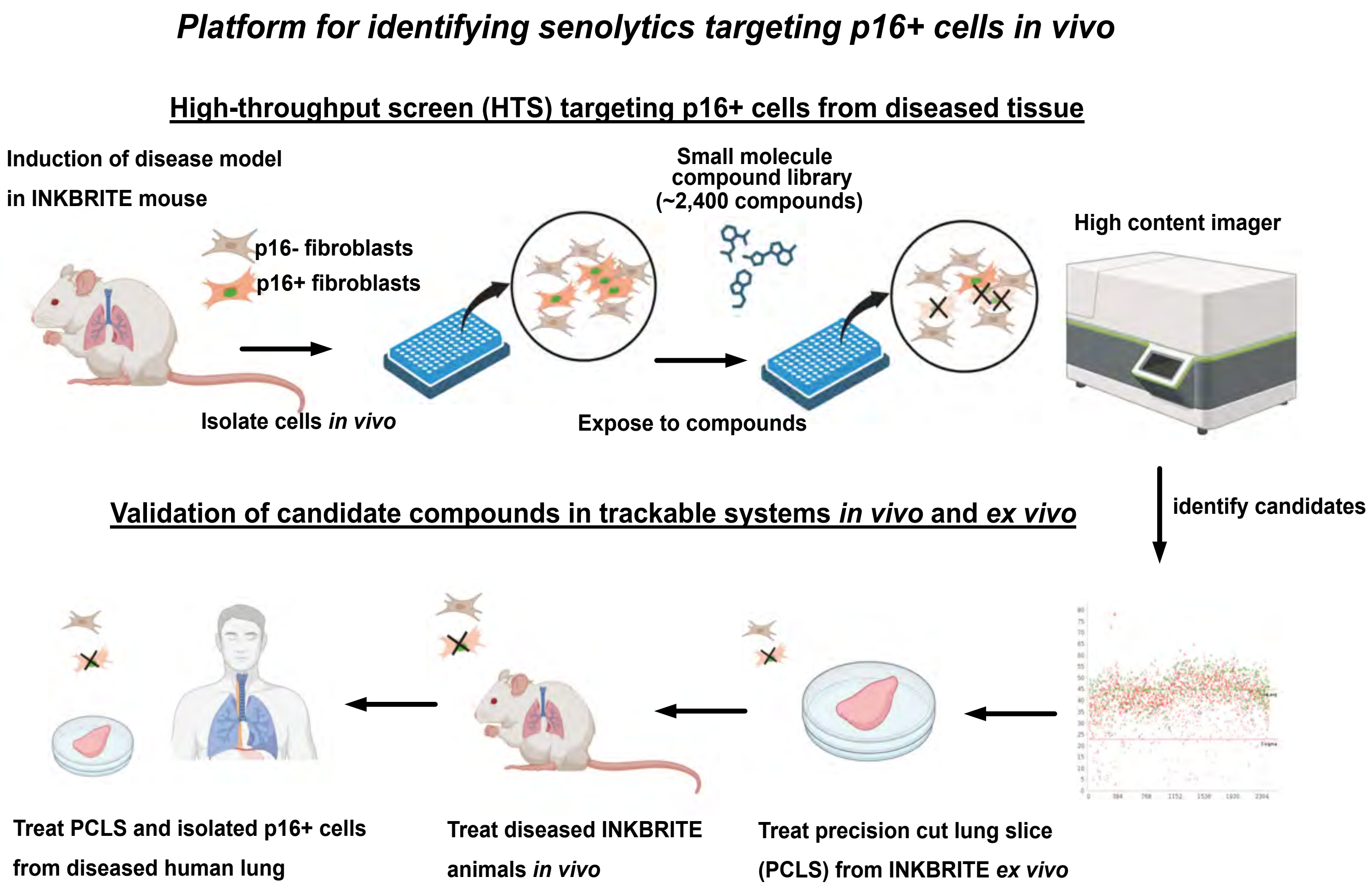

Platform for identifying senolytics targeting p16+ cells in vivo
High-throughput screen (HTS) targeting p16+ cells from diseased tissue
Induction of disease model in INKBRITE mouse
p16- fibroblasts
p16+ fibroblasts
Isolate cells in vivo
Small molecule compound library (~2,400 compounds)
Expose to compounds
High content imager
identify candidates
Validation of candidate compounds in trackable systems in vivo and ex vivo
Treat precision cut lung slice (PCLS) from INKBRITE ex vivo
Treat diseased INKBRITE animals in vivo
Treat PCLS and isolated p16+ cells from diseased human lung
